# Progerin impairs 3D genome organization and induces fragile telomeres by limiting the dNTP pools

**DOI:** 10.1038/s41598-021-92631-z

**Published:** 2021-06-23

**Authors:** Anna Kychygina, Marina Dall’Osto, Joshua A. M. Allen, Jean-Charles Cadoret, Vincent Piras, Hilda A. Pickett, Laure Crabbe

**Affiliations:** 1 Molecular, Cellular and Developmental Biology Department (MCD), Centre de Biologie Integrative (CBI), CNRS, UPS, University of Toulouse, 31062 Toulouse, France; 2 Telomere Length Regulation Unit, Faculty of Medicine and Health, Children’s Medical Research Institute, University of Sydney, Westmead, NSW 2145 Australia; 3 Université de Paris, CNRS, Institute Jacques Monod , F-75006 Paris, France; 4 INSERM UMR1291, CNRS UMR5051, UT3, Toulouse Institute for Infectious and Inflammatory Diseases (Infinity), 31059 Toulouse, France

**Keywords:** Nuclear envelope, Nuclear organization, Senescence, Telomeres

## Abstract

Chromatin organization within the nuclear volume is essential to regulate many aspects of its function and to safeguard its integrity. A key player in this spatial scattering of chromosomes is the nuclear envelope (NE). The NE tethers large chromatin domains through interaction with the nuclear lamina and other associated proteins. This organization is perturbed in cells from Hutchinson–Gilford progeria syndrome (HGPS), a genetic disorder characterized by premature aging features. Here, we show that HGPS-related lamina defects trigger an altered 3D telomere organization with increased contact sites between telomeres and the nuclear lamina, and an altered telomeric chromatin state. The genome-wide replication timing signature of these cells is perturbed, with a shift to earlier replication for regions that normally replicate late. As a consequence, we detected a higher density of replication forks traveling simultaneously on DNA fibers, which relies on limiting cellular dNTP pools to support processive DNA synthesis. Remarkably, increasing dNTP levels in HGPS cells rescued fragile telomeres, and improved the replicative capacity of the cells. Our work highlights a functional connection between NE dysfunction and telomere homeostasis in the context of premature aging.

## Introduction

Telomeres are specialized nucleoprotein complexes capping the ends of linear chromosomes. They consist of repetitive nucleotide sequences (-TTAGGG- in mammals) that end with a 3′-overhang, and are shielded by a specific protein complex called shelterin^1,2^. The primary function of telomeres is to delineate chromosome ends and avoid illicit DNA repair that would result in chromosome fusions and subsequent genomic instability and aneuploidy^3,4^. Shelterin plays a major role in repressing the DNA damage response at chromosome ends. Consistently, the depletion or the loss of function of shelterin components leads to the activation of ATM- or ATR-dependent DNA damage responses, cell cycle arrest, and chromosome instability^5^. Another essential function of telomeres is to protect chromosome ends from DNA resection occurring after each round of replication due to the end replication problem. Therefore, telomeres from human somatic tissue shorten during log-phase cell growth, leading to a progressive change in their structure that entails replicative senescence^4,6^. Restricting the growth of aged cells serves as a tumor suppressor mechanism, but also contributes to cellular and organismal aging^7^. Telomere shortening can be counteracted by the activity of telomerase, the enzyme responsible for telomere elongation. In telomerase-positive cells such as stem cells and germline cells, telomeres are maintained to a stable length, bypassing senescence and resulting in cellular immortalization^8^.

The connection between telomere length and human organismal aging is highlighted by a large panel of age-related human syndromes associated with short telomeres and signs of premature senescence^9^. Premature aging disorders are usually caused by mutations impairing the DNA damage response and repair pathways (Ataxia Telangiectasia), shelterin complex structure (Dyskeratosis Congenita), and telomere length regulation (Dyskeratosis Congenita, Bloom and Werner Syndromes). These telomere-related syndromes share overlapping traits with human syndromes linked to lamins, grouped under the term of laminopathies^10^. In metazoans, the lamina forms a thin meshwork lining the inner side of the nuclear envelope (NE) and is composed of a family of proteins consisting of A-type and B-type lamins. In addition to a structural function of the lamina in maintaining the shape and the mechanical properties of the nucleus, there is growing evidence suggesting that nuclear lamins also play a major role in genome organization and stability^11^. One of the most severe laminopathies, Hutchinson-Gilford progeria syndrome (HGPS), is predominantly caused by a de novo point mutation G608G (nucleotide 1824 C > T) in exon 11 of the *LMNA* gene encoding both LaminA and C isoforms. This leads to the expression of an aberrantly spliced and processed protein termed progerin^10,12^, causing premature aging at the organismal level. Compared to normal fibroblasts, the growth potential of cultured HGPS fibroblasts is strongly impaired. They also display a low but persistent activation of DNA damage checkpoints, and carry short telomeres^13–19^. One key feature of HGPS cells is their abnormal nuclear architecture, with lobulation of the NE, thickening of the nuclear lamina, and defects in nuclear pore organization^20^. The NE represents a main organizer of chromatin within the 3D nuclear space. During the last decade, it has become clear that the position of chromatin in the nucleus impacts genome stability and gene regulation^21,22^. Large heterochromatin domains are anchored to the nuclear lamina to form the so-called LADs (lamina-associated domains), regions that are essentially transcriptionally repressed and known to replicate late^23–25^. The nuclear architecture defects observed in progerin-expressing cells have dramatic consequences on chromatin organization, with a loss of some heterochromatin-lamina domains, decreased interactions within heterochromatin domains, and epigenetic alterations in LADs^26–28^.

How these changes in 3D chromatin organization account for the proliferative defects of HGPS cells remains unclear. Telomere maintenance is a key factor, as telomere elongation by telomerase expression prevents progerin-dependent growth defects^17,29^. In fact, several lines of evidence indicate that telomeres and lamins are directly connected, and that nuclear substructures impact telomere function and stability. In interphase cells, human telomeres are organized close to the center of the nucleus^30,31^, but a few late-replicating telomeres are anchored to the NE^32,33^. In contrast, a large subset of telomeres is tethered to the NE during post-mitotic nuclear assembly^34^, in close proximity to Lamin B1^35^. Connection between telomeres and lamins also occurs in the nucleoplasm and plays a role in telomere protection^36^.

In this study, we further explored the interconnection between telomere maintenance, lamin-dependent nuclear organization, and aging. We demonstrate that progerin expression perturbs telomere 3D organization and alters their chromatin state. Telomere replication is challenged, leading to an accelerated telomere shortening that limits cell growth. We also linked the replication defect to nuclear organization by studying the replication timing (RT) signature of progerin-expressing cells. A significant fraction of the genome indeed shifted to earlier replication, in agreement with the loss of peripheral heterochromatin that characterizes HGPS. Finally, we uncovered that dNTP pools seem limiting in progerin cells, as supplementation with nucleosides restored their replication competence and alleviated the telomere replication stress. We propose that an alteration of the 3D nuclear architecture of HGPS cells modifies their replication timing program, leading to a higher density of active replication forks that limits dNTPs availability and promotes fork stalling. We show that telomeres are particularly sensitive to this replication defect, and the resulting short telomeres trigger premature growth arrest.

## Results

### Progerin impairs 3D-telomere organization in human primary skin fibroblasts

**Figure 1:**
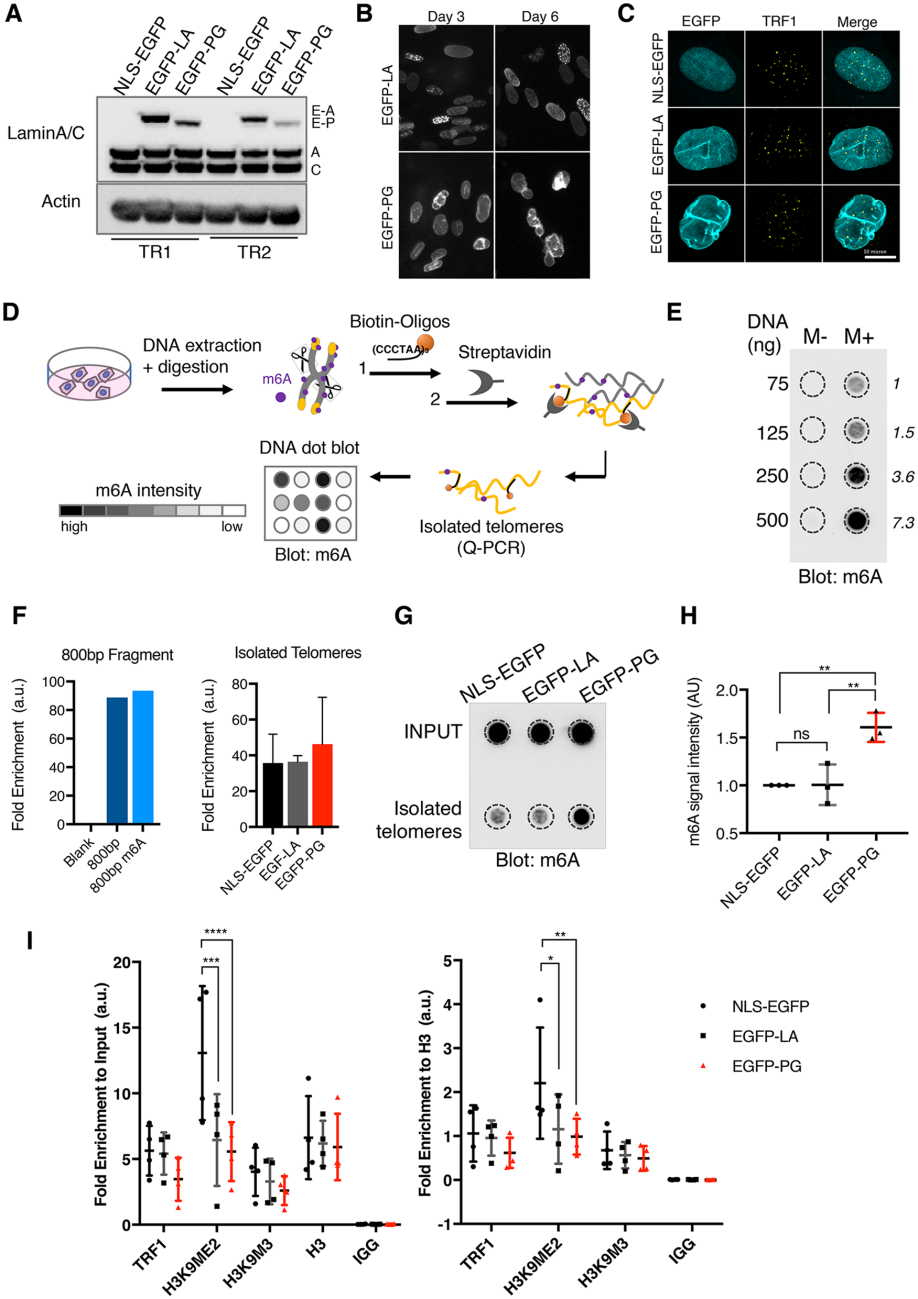
Progerin impairs 3D telomere organization. (**A**) Western blots of whole cell extracts of HDF cells expressing the indicated constructs. Two independent transductions are shown (TR1 and TR2). The membrane was probed with the LaminA/C antibody to detect endogenous LaminA (A), endogenous LaminC (C) and the EGFP-fusion proteins EGFP-LA (E-A) and EGFP-PG (E-P). Actin is shown as a loading control. (**B**) Live HDF cells expressing EGFP-LA or EGFP-PG, 3 or 6 days post-transduction. The EGFP signal (grey) is detected using a benchtop fluorescent microscope. (**C**) Representative immunostaining of HDF cells expressing the indicated constructs. EGFP (cyan), TRF1 (yellow), and merge. The max projection is shown. Scale bar: 10 μm. (**D**) Scheme of MadID-dotblot principle. Genomic DNA is extracted from cultured cells, and digested with frequent cutter restriction enzymes that do not cut the telomeres. Telomeres are isolated using biotinylated oligonucleotides complementary to the TTAGGG telomeric sequence and streptavidin beads. The purity and the amount of isolated telomeres are verified by qPCR, before they are blotted on a membrane for hybridization with a m6A-specific antibody. (**E**) Dotblot of increasing amount of lambda DNA isolated from Dam− (M−) or Dam+ (M+) bacterial strains. The membrane is hybridized with a m6A-specific antibody. The m6A signal intensity in the M+ DNA is indicated on the right, relative to the signal obtained with 75 ng of lambda DNA. (**F**) qPCR analyses of MadID using TelC/TelG primers to amplify telomeric DNA. Left panel: a fragment of 800 bp of telomeric repeats was used as a template, before or after in vitro methylation with M.EcoGII. Right panel: isolated telomeres from HDF expressing NLS-EGFP, EGFP-LA, or EGFP-PG as indicated. Enrichments are shown for three independent telomere purifications. Fold enrichments are calculated against INPUTs, i.e. genomic DNA. (**G**) Representative example of a MadID-dotblot experiment performed on HDF cells expressing NLS-EGFP, EGFP-LA and EGFP-PG. INPUT DNA and isolated telomeres were blotted on a membrane hybridized with a m6A-specific antibody. (**H**) Quantification of 3 independent MadID experiments similar to (**E**). The graph represents the normalized m6A intensity (normalized to the amount of isolated telomeres measured by qPCR) relative to the EGFP-NLS control. Mean with SD is shown, n = 3. Statistical significance was determined using one-way Anova (**indicates p < 0.01). (**I**) ChIP analyses on HDF cells expressing NLS-EGFP, EGFP-LA, and EGFP-PG. Immunoprecipitations were performed with the indicated antibodies. The graphs represent the enrichment relative to input material (left) or to H3 (right) (Mean ± SD, n = 4). Statistical significance was determined using two-way Anova (*indicates p < 0.05, **indicates p < 0.01, ***indicates p < 0.001, ****indicates p < 0.0001).

The impact of A-type lamins on telomere organization was previously investigated in *Lmna*^*−/−*^ mouse cells, where a significant change in distribution of telomeres toward the nuclear periphery was observed^37^. We hypothesized that the expression of progerin in HGPS cells may also affect human telomere organization and be detrimental for their maintenance. To model the physiopathology of HGPS fibroblasts, we established normal Human Dermal Fibroblasts (HDF) cell lines stably expressing EGFP-tagged human LaminA∆50/progerin (EGFP-PG), and used wild type LaminA (EGFP-LA) or a nuclear localized EGFP (NLS-EGFP) as controls (Fig. 1A, Supplemental Fig. S1A). Within 3 days post-transduction, progerin expression led to strongly misshapen nuclei (Fig. 1B). In contrast, wild-type LaminA was also properly integrated into the lamina but did not affect nuclear morphology. Some nuclei from EGFP-LA cells displayed additional foci in the nucleoplasm, which correspond to LaminA precursors before their processing^38^. EGFP-PG expression resulted in previously reported phenotypes such as mislocalization of the protein SUN1^39^, as well as a decreased expression of LAP2alpha, H3K9me3, H3K27me3, and LaminB1^40^ (Fig. S1B–E). Expression of EGFP-LA also impacted LAP2alpha levels (Fig. S1C,D) as previously described^41^. To assess the impact of progerin on telomere distribution in the nuclear volume, we imaged telomeres by immunostaining of the TRF1 shelterin protein (Fig. 1C). However, the extent of NE blebbing with deep membrane invaginations reaching the nucleus center made it difficult to evaluate telomere localization with regards to the nuclear lamina by microscopy. To solve this issue, we applied MadID, a proximity labeling technique that we recently developed to probe telomere-NE contact sites^35,42^. MadID relies on the expression of a bacterial methyltransferase called M.EcoGII, which catalyzes the methylation of adenine residues (m6A). Fused to a protein of interest, adenines within and in the vicinity of the protein binding sites will be decorated with m6A, a modification that can be easily detected using a m6A-specific antibody. We previously fused M.EcoGII to LaminB1 to probe chromatin-NE contact sites, and were able to map LADs with high specificity, resolution and genome coverage^35^. This experiment also revealed telomeres and NE contact sites in normal and cancer cells. We therefore performed MadID-dotblot as previously described^42^ (Fig. 1D). Briefly, intact telomeric repeats were released by restriction enzymes digestion and purified using biotin-(CCCTAA)_3_ oligos and streptavidin-coated magnetic beads. Input DNA and purified telomeric DNA samples were next blotted and probed with a m6A-specific antibody. We confirmed the dynamic range of our assay by blotting increasing amounts of lambda DNA obtained from *dam*+ (with methylation) or *dam− *(no methylation) *E. coli* strains. The resulting m6A signal was specific and proportional to the amount of DNA (Fig. 1E). To implement MadID in our setting, M.EcoGII-LaminB1 was expressed using an inducible retroviral vector in our established HDF cell lines. We confirmed the proper expression of M.EcoGII-LaminB1 fusion protein 24 h after induction, with a level of induction comparable in HDF NLS-EGFP, EGFP-LA, and EGFP-PG (Fig. S1F). These cells were used to purify telomeric DNA. The efficiency of telomere capture and purity was verified by qPCR, based on an amplification method previously established to measure telomere length^43^. A known concentration of a fragment of 800 bp of TTAGGG repeats was used to verify that qPCR efficiency was close to 100%. This fragment was also in vitro methylated by recombinant M.EcoGII^35^ in order to assess the impact of m6A residues on PCR amplification. We did not detect any differences in fold enrichment with or without m6A methylation, confirming that qPCR is a suitable approach to assess the amount of purified telomeres in HDF samples, regardless of their methylation status (Fig. 1F-left panel). We successfully purified telomeres from our HDF samples (Fig. 1F-right panel) and confirmed that they did not contain detectable genomic DNA using single copy gene amplification^43^ (Fig. S1G). Analysis of the resulting blots revealed that M.EcoGII-LaminB1 was able to methylate genomic DNA in NLS-EGFP, EGFP-LA and EGFP-PG cells (Fig. 1G, see Input samples). m6A signal was also detectable on isolated telomeres for all three samples, which indicates that a subset of telomeres contact the nuclear lamina in asynchronous HDF cells^35^. Interestingly, progerin expression induced a 1.5 fold increase in m6A signal at telomeres (Fig. 1G,H). This result suggests that telomeres contact the lamina more frequently in progerin-expressing cells. Changes in 3D-chromatin environment could impact telomere chromatin structure, such as the establishment of heterochromatin marks. This is particularly relevant in the context of HGPS patients, since HGPS fibroblasts show an alteration of constitutive heterochromatin histone marks. We carried out ChIP experiments using H3K9me2 and H3K9me3 specific antibodies, as well as TRF1 and H3 as internal controls. We observed a marked decrease of H3K9me2 at telomeres in progerin cells, when signals were normalized to Input or H3 levels (Fig. 1I). Similarly, although not statistically significant, we reproducibly found that levels of H3K9me3 were also lower. Overall, these results indicate that progerin expression affect telomere 3D organization as well as their chromatin state, in line with the global loss of heterochromatin previously reported in HGPS cells. Interestingly, expression of wild-type LaminA also decreased the levels of H3K9me2 and me3 levels at telomeres. This suggests that a modification in the LaminA/Lamin B1 balance is detrimental for chromatin maintenance at telomeres.

### Progerin impairs telomere length maintenance and induces premature senescence in primary skin fibroblasts

**Figure 2:**
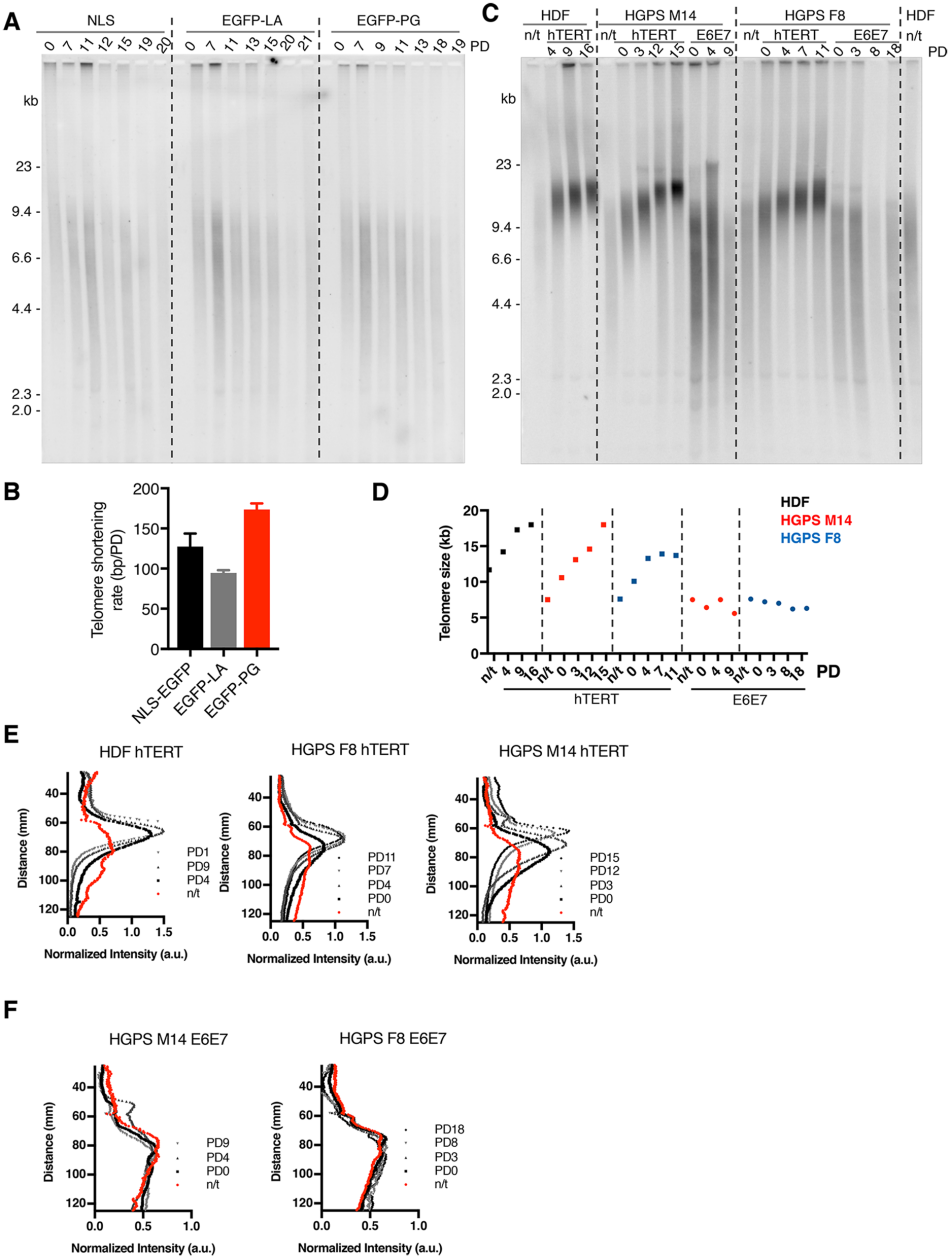
Critically short telomeres induce senescence in progerin-expressing cells. (**A**) Southern blot of terminal restriction fragments (TRF) of HDFs expressing NLS-EGFP, EGFP-LA and EGFP-PG. The cumulative population doublings (PD) are indicated. A representative exemple is shown from n = 2 experiments. (**B**) Telomere shortening rate quantified from two independent TRF analyses as in (**A**). Mean with SD is shown. (**C**) Southern blot of terminal restriction fragments (TRF) from normal HDF, HGPS M14, HGPS F8 cells before and after expression of hTERT or E6E7 (n = 1). The cumulative population doublings (PD) are indicated. (**D**) Quantification of TRF analyses from (**C**). The graph represent the telomere size in kb in the different cell lines with cumulative PDs. (**E,F**) Quantification of TRF analyses from (**C**) before (n.t.) or after hTERT expression (**E**) or E6E7 expression (**F**). The graphs represent the normalized signal intensity as a function of the distance on the gel, a proxy of telomere length.

Next, we aimed to determine whether these changes in 3D telomere organization and chromatin state correlate with defects in telomere length maintenance. HDF cell lines expressing EGFP-NLS, EGFP-LA and EGFP-PG were cultured until they reached their growth plateau. Physiological oxygen conditions (5%) were used to limit oxidative stress that strongly affects the fitness of progerin-expressing cells^44^. Remarkably, although ectopic expression of progerin instantly affected NE integrity (Fig. 1B), several population doublings were necessary to visualize a change in the growth rate (Fig. S2A,B). We consistently observed a growth defect in progerin-expressing cells, together with an early detection of senescent cells that accumulated over time (Fig. S2C). Using southern blots of terminal restriction fragments (TRFs), we first confirmed that ectopic expression of progerin accelerated the rate of telomere shortening^45^ (Fig. 2A). Telomeres shortened at a rate of 100–150 bp/PD in NLS-EGFP and EGFP-LA control HDFs, which is comparable to what has been described in other primary fibroblasts^46^ (Fig. 2B). In contrast, telomeres shortened 1.5-times faster in EGFP-PG cells. Importantly, we did not observe an acceleration of telomere shortening after wild-type LaminA expression, unlike what was previously described^45^. As critically short telomeres are the trigger of senescence entry in primary fibroblasts, these results suggest that the presence of short telomeres in HGPS cells is responsible for their premature senescence. Consistent with this idea, telomere elongation by telomerase expression was shown to rescue the growth defect of progerin-expressing cells^17,29,47,48^. Other studies suggested that telomerase fails to immortalize HGPS cells without the concomitant repression of p53. Indeed, hTERT expressing HGPS cells still reached senescence, while normal fibroblasts were immortalized^49,50^. To investigate this further, we assessed the growth potential and telomere length of HGPS patients skin fibroblasts obtained from Coriell Cell Repositories (see “Method” section for further details). Both HGPS M14 and HGPS F8 cell lines grew very poorly (Fig. S2D), with senescent cells present in the cell population at the onset of culturing (Fig. S2E). HGPS M14 cells presented the strongest growth defects, with up to 75% of senescent cells. Senescence entry was due to checkpoint activation, since suppression of pRB and p53 pathways by expression of the human papilloma virus serotype 16 (HPV16) E6 and E7 oncoproteins allowed HGPS cells to bypass senescence (Fig. S2D,E). We confirmed that initial telomere length was much shorter in both patient cells (6–7 kb) than in control HDFs from healthy donors (< 9 kb)^13,16,48^ (Fig. 2C). In our hands, expression of the catalytic subunit of telomerase hTERT rescued the growth potential of these HGPS cell lines, even in the presence of functional checkpoints (Fig. S2D). hTERT also increased the percentage of BrdU positive cells (Fig. S2F,G). The growth advantage driven by telomerase was stronger for HGPS M14 compared to HGPS F8, with an increase from 9% to about 18% of S phase cells after hTERT expression, in agreement with the greater growth defect initially observed and higher percentage of senescence cells. Interestingly, hTERT expression in HGPS M14 cells also increased laminB1 levels, known to decrease upon senescence (Fig. S2H). Shortly after hTERT expression, telomere length increased in both HGPS cells, with an efficiency of telomere elongation comparable to HDF (Fig. 2D,E). Telomere length was then stabilized similarly in patients and healthy cells, suggesting that progerin did not impair the regulation of telomere elongation by telomerase^51^. Telomere length changed upon E6–E7-dependent checkpoint inactivation in M14 cells, with a wider range of size reaching very short (2 kb) and very long (more than 10 kb) telomeres (Fig. 2C-F). These results indicate that critically short telomeres are a trigger for growth arrest in progerin-expressing cells, and their elongation is sufficient to prevent checkpoint-induced senescence.

### Progerin expression induces fragile telomeres in human cells

**Figure 3:**
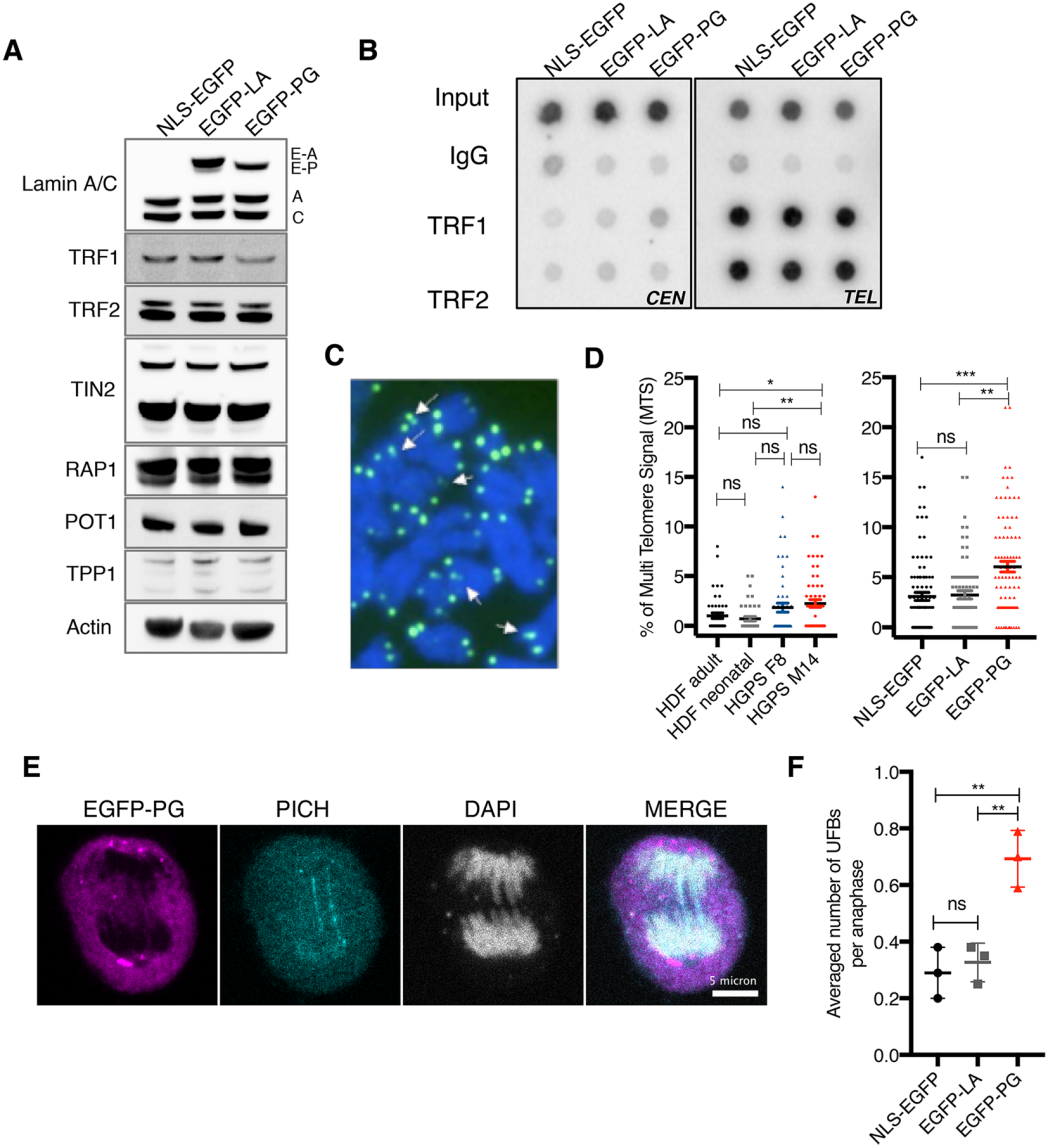
Progerin challenges telomere replication. (**A**) Western blots of whole cell extracts of the indicated cell lines. Antibodies used are indicated. A representative actin loading control is shown. (**B**) Representative ChIP-dot blot on HDF cells expressing NLS-EGFP, EGFP-LA or EGFP-PG. Immunoprecipitations were performed with the indicated antibodies and dot blots hybridized with telomere (TEL) or centromere (CEN)-specific probes. (**C**) Representative image of a metaphase spread from HDF cells expressing EGFP-PG. Telomeric DNA is visualized with a PNA probe (FITC) and DNA is stained with DAPI. Arrows point to chromosome ends with Multiple Telomere Signal (MTS). (**D**) Quantification of MTS in HGPS cells and controls (left) or HDF expressing the indicated constructs (right). The graphs represent the percentage of MTS per metaphase ± SD (n = 3). A total of 73 to 94 metaphases were scored in HDF cells, and between 42 and 58 metaphases in HGPS cells. Statistical significance was determined using Mann Whitney test (*indicates p < 0.05, and **p < 0.005). (**E**) Representative immunostaining of HDF cells expressing EGFP-PG. EGFP signal (purple), PICH (cyan), and DAPI (grey). Scale bar: 5 μm. (**F**) Quantification of PICH immunostaining as in C for HDF cells expressing NLS-EGFP, EGFP-LA, EGFP-PG. The graph represents the average number of ultra-fine bridges (UFBs) per anaphase in the indicated cell lines (with SD). Statistical significance was determined using unpaired t-test, n = 3 (**indicates p < 0.005).

While the impact of progerin expression on telomere length was previously suggested, the mechanism of action is unknown. To address this key question, we monitored telomere integrity in EGFP-PG, EGFP-LA and NLS-EGFP cells. First, we observed that the expression of the six subunits of the shelterin complex in whole cell extracts was unaffected by ectopic progerin expression (Fig. 3A, Supplemental Fig. S3A). In addition, ChIP experiments confirmed that both TRF1 and TRF2, the core shelterin proteins that directly bind double stranded TTAGGG repeats, were correctly addressed at telomeres in EGFP-PG expressing cells (Fig. 3B, Supplemental Fig. S3B). Telomere integrity was therefore analyzed on metaphase spreads prepared from HGPS patient cells or HDF cells expressing NLS-EGFP, EGFP-LA and EGFP-PG. As HGPS patient cells have a low mitotic index, cells were pre-synchronized by a double thymidine block, released for 7 h and treated with colcemide to arrest them in metaphase by microtubule depolymerization. To visualize telomeres, metaphase chromosomes were stained with a telomere specific PNA FISH probe fused to FITC. Telomere aberrations, such as telomere loss, sister chromatid fusions, telomere fragments, and end-to-end telomere fusions were screened using this method but no significant difference was observed compared to control cells (Fig. S3C). However, fragile telomeres, telomeric regions where replication is impaired and that are visualized as multi-telomere signals (MTS)^52,53^, were found at both progerin-expressing HGPS cell lines (Fig. 3C,D). Interestingly, this phenotype was more pronounced in HGPS M14 compared to HGPS F8, indicating patient-to-patient variability and a possible link to the age of the donor. We also found a significant increase of MTS in HDF expressing EGFP-PG compared to controls (Fig. 3D). These results indicate that telomere replication is affected in progerin-expressing cells, which explain their length maintenance defect and the rescue by telomerase elongation. This prompted us to assess the presence of ultrafine anaphase bridges (UFBs), residual secondary structures observed in anaphase that must be processed to ensure faithful chromosome segregation. One class of UFBs arises from under-replicated intermediate structures, often associated with fragile sites at genome regions difficult to replicate. UFBs can be identified indirectly by immunostaining of the resolvase PICH that binds UFBs to promote their dissolution at the onset of mitosis^54^. NLS-EGFP, EGFP-LA and EGFP-PG HDF were synchronized with a single thymidine block to enrich the cell population in anaphase cells. At this stage, the diffuse EGFP staining reflected NE breakdown that occurs during open mitosis and releases lamins (Fig. 3E). PICH staining was also diffuse in cells with no UFBs, but formed thread-like structures when UFBs were present (Fig. 3E). We found that the number of UFBs per anaphase was significantly higher in progerin cells compared to controls (Fig. 3F). Altogether, our results point to a DNA replication defect triggered by progerin expression that affects telomere replication.

### Progerin expression sensitizes cells to replication stress

**Figure 4:**
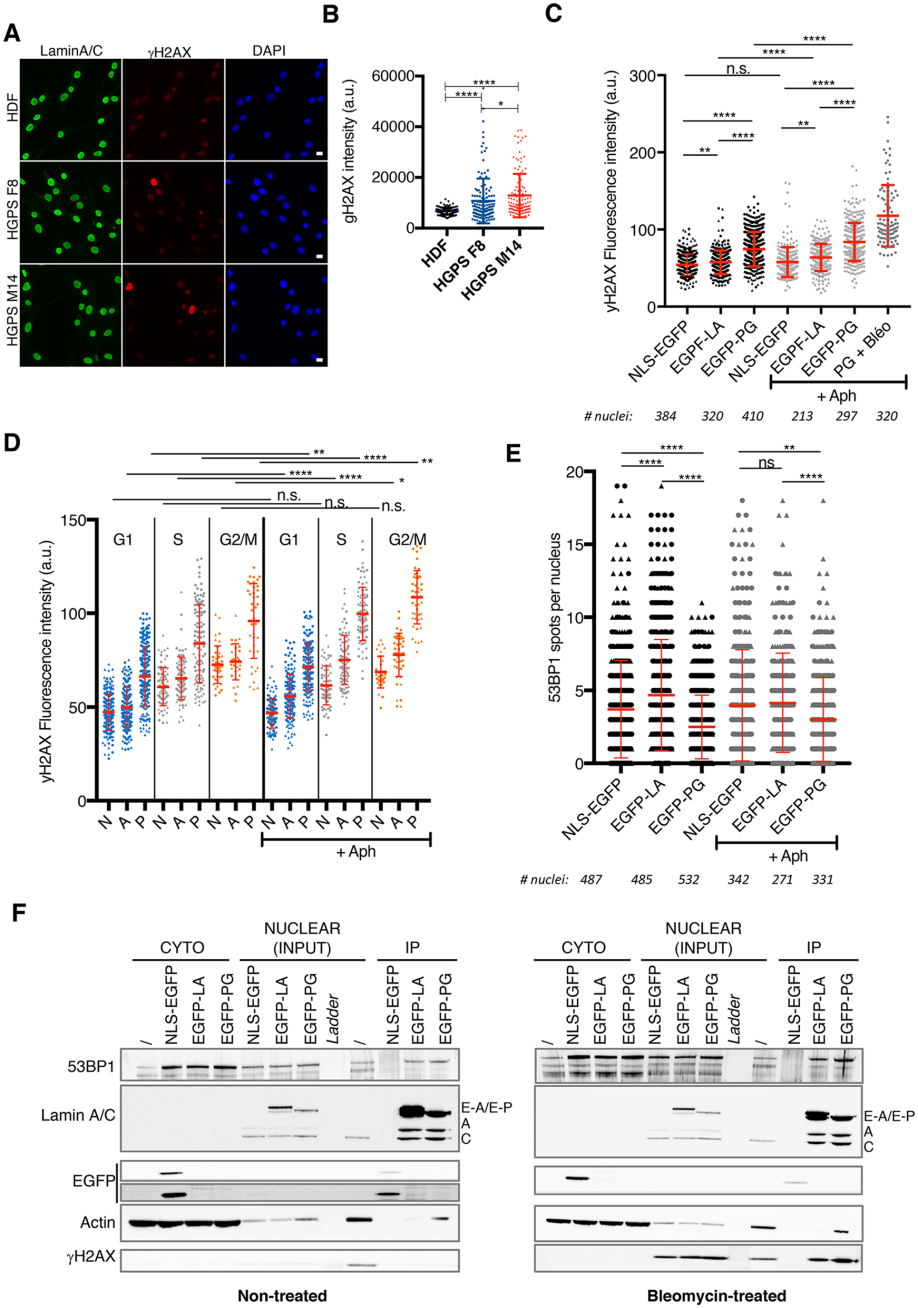
Progerin expression sensitizes cells to replication stress. (**A**) Representative immunostaining of γH2AX in HGPS and HDF cells. LaminA/C (green), γH2AX (red), and DAPI (blue). Scale bar: 5 μm. (**B**) Quantification of (**A**). The graph represents the γH2AX mean signal intensity ± SD. Statistical significance was determined using unpaired t-test (*indicates p < 0.05, and ****p < 0.0001). At least 100 cells were analyzed per condition, n = 1. (**C,E**) Quantification of QIBC for γH2AX (**C**) or 53BP1 (**E**) staining performed on HDF expressing NLS-EGFP, EGFP-LA and EGFP-PG treated or not with 0.2 μM of aphidicolin. The graph represents γH2AX mean signal intensity (**C**) or the number of 53BP1 foci (**E**) in the different conditions. Two independent transductions of the constructs were performed in HDF cells and are displayed with different symbols (triangles and circles, respectively). Each symbol corresponds to a single nucleus. The number of nuclei scored is indicated at the bottom of the graphs. Mean with SD is shown, n = 2. Statistical significance was determined using the Mann–Whitney test on the pool of cells (**indicates p < 0.05, ****indicates p < 0.0001). (**D**) Quantification of QIBC for γH2AX staining performed on HDF expressing NLS-EGFP (N), EGFP-LA (A) and EGFP-PG (P) treated or not with 0.2 μM of aphidicolin as in (**C**). Nuclei were sorted in the different phases of the cell cycle (G1, S and G2/M) according to the DAPI and EdU signals. Mean with SD is shown, n = 2. Statistical significance was determined using Mann–Whitney test on the pool of cells (*indicates p < 0.05, **indicates p < 0.005, ****indicates p < 0.0001). (**F**) Co-immunoprecipitation experiment of HDF cells expressing NLS-EGFP, EGFP-LA and EGFP-PG before (non-treated, left panel) or after bleomycin treatment of 10 μg/ml, 30 min (bleomycin-treated, right panel) to induce DNA damage. Immunoprecipitations were performed on nuclear extracts. The western blots display the cytoplasmic fraction, the nuclear fraction corresponding to the INPUT material, and the immunoprecipitated fraction (IP). Membranes were probed with the indicated antibodies. Actin is used as a control for cytoplasmic fraction, while both LaminA/C and γH2AX are strongly enriched in the nuclear fraction. The LaminA/C reveals endogenous levels of LaminA, LaminC, and EGFP-fusion constructs (respectively labeled A, C, E-A, E-P). n = 1.

Replication defects impact DNA integrity and induce endogenous checkpoint activation and DNA damage responses. Several studies reported an increase of the DNA damage markers γH2AX and 53BP1 in mouse models defective for LaminA processing, in HGPS cells, and in normal cells following ectopic expression of progerin^15,17,19,44^. This was observed in the absence of exogenous DNA damage, suggesting that A-type lamin integrity can affect chromatin maintenance. Indeed, we confirmed that endogenous levels of γH2AX were higher in both HGPS cell lines compared to normal HDFs (Fig. 4A,B). To assess the impact of replicative stress on the level of endogenous DNA damage, we performed quantitative image based cytometry (QIBC) after ectopic progerin expression, using EdU incorporation combined with γH2AX and 53BP1 immunostaining^55,56^. QIBC allows to simultaneously assess cell-cycle distribution, replication competence, and DNA damage signaling. γH2AX was found enriched in HDF expressing EGFP-PG compared to controls (Fig. 4C), with a higher intensity in S and G2/M phases of the cell cycle (Fig. 4D). When DNA replication was challenged with 0.2 μM of aphidicolin for 24 h, we observed an accumulation of cells in S phase in all three cell types, but more pronounced for LaminA- and progerin-expressing cells (Fig. S4A). This treatment did not induce a significant increase of γH2AX in EGFP-NLS control cells (Fig. 4C,D). However, γH2AX staining was strongly increased in progerin cells, pointing to their sensitivity to replication stress. The accumulation of γH2AX was evident in S phase, but foci also accumulated in G1 and G2/M, arguing for a transmission of the damage signaling outside of S phase (Fig. 4D). We noticed that expression of wild-type LaminA also perturbed chromatin integrity, although to a lower extent compared to the progerin mutant. Interestingly, scoring 53BP1 spots in these cells yielded in a completely different outcome. Although endogenous levels of 53BP1 foci were detected in HDF NLS-EGFP and EGFP-LA, a drastic drop of the number of foci was consistently observed in EGFP-PG cells (Fig. 4E, Supplemental Fig. S4B–D). These results confirm the defective recruitment of 53BP1 to sites of DNA damage that was previously described in progerin cells^14^. Interestingly, the induction of 53BP1 DNA damage foci was observed after a short term induction of Progerin expression using an inducible expression system^17^. This suggests that Progerin effect on the DNA damage response might require longer expression periods, and is not an immediate effect. In undamaged normal fibroblasts, 53BP1 binds to A-type lamins and this interaction was proposed to serve as a storage to facilitate 53BP1 recruitment to sites of DNA damage^57^. Indeed, in absence of functional LaminA, 53BP1 is degraded by a cysteine protease^58^. Although the pool of functional LaminA is impaired in HGPS patient, it remains unclear how progerin expression impacts 53BP1 and its recruitment to DNA damage sites. To test this, we performed co-immunoprecipitation experiments using EGFP pull-down in HDF NLS-EGFP, EGFP-LA and EGFP-PG, before and after bleomycine treatment to induce DNA damage. In order to improve 53BP1 protein recovery and the sensitivity of our assay, cell extracts were fractionated to separate the cytoplasmic fraction, here enriched in actin, and the nuclear fraction enriched in LaminA/C (Fig. 4F, see INPUTs). After bleomycin treatment, γH2AX was detected in the nuclear fraction, confirming the checkpoint activation following induced DNA damage. Pull-downs were performed on nuclear extracts only, and IP fractions clearly recovered EGFP-LA and EGFP-PG (Fig. 4F, see E-A and E-P bands). Consistent with the fact that LaminA interacts with itself and with progerin, endogenous LaminA/C bands were also detected in the IP lanes (Fig. 4F, see A and C bands). 53BP1 was found in both EGFP-LA and EGFP-PG pull-downs, but not in the control fraction NLS-EGFP, regardless of the bleomycin treatment. This confirms the specificity of the 53BP1-LaminA interaction, and indicates that progerin expression did not prevent this interaction. LaminA-53BP1 interaction is supposed to decrease upon DNA damage, in order to release 53BP1 that can be addressed to sites of damage^57^. We did not detect a clear drop in 53BP1 signal after bleomycin treatment in our LaminA IP fraction. However, progerin IP did retrieve 53BP1 efficiently, suggesting that progerin could sequester the DNA damage marker and prevent its recruitment to sites of DNA damage. Our results suggest that progerin cells are sensitive to drugs challenging DNA replication. A low but persistent DNA damage checkpoint is activated, but the pathway is impaired due to a decreased recruitment of 53BP1. Previous work reported that the DNA damage response in HGPS originates from telomeres^47^. In our hands, DNA damage markers did not colocalize with telomeres using TRF1 co-staining in immunofluorescence, and we could not detect γH2AX at telomeric repeats using ChIP. This is in accordance with the work by Wheaton et al.^49^.

### Progerin expression perturbs replication fork progression at telomeres

**Figure 5:**
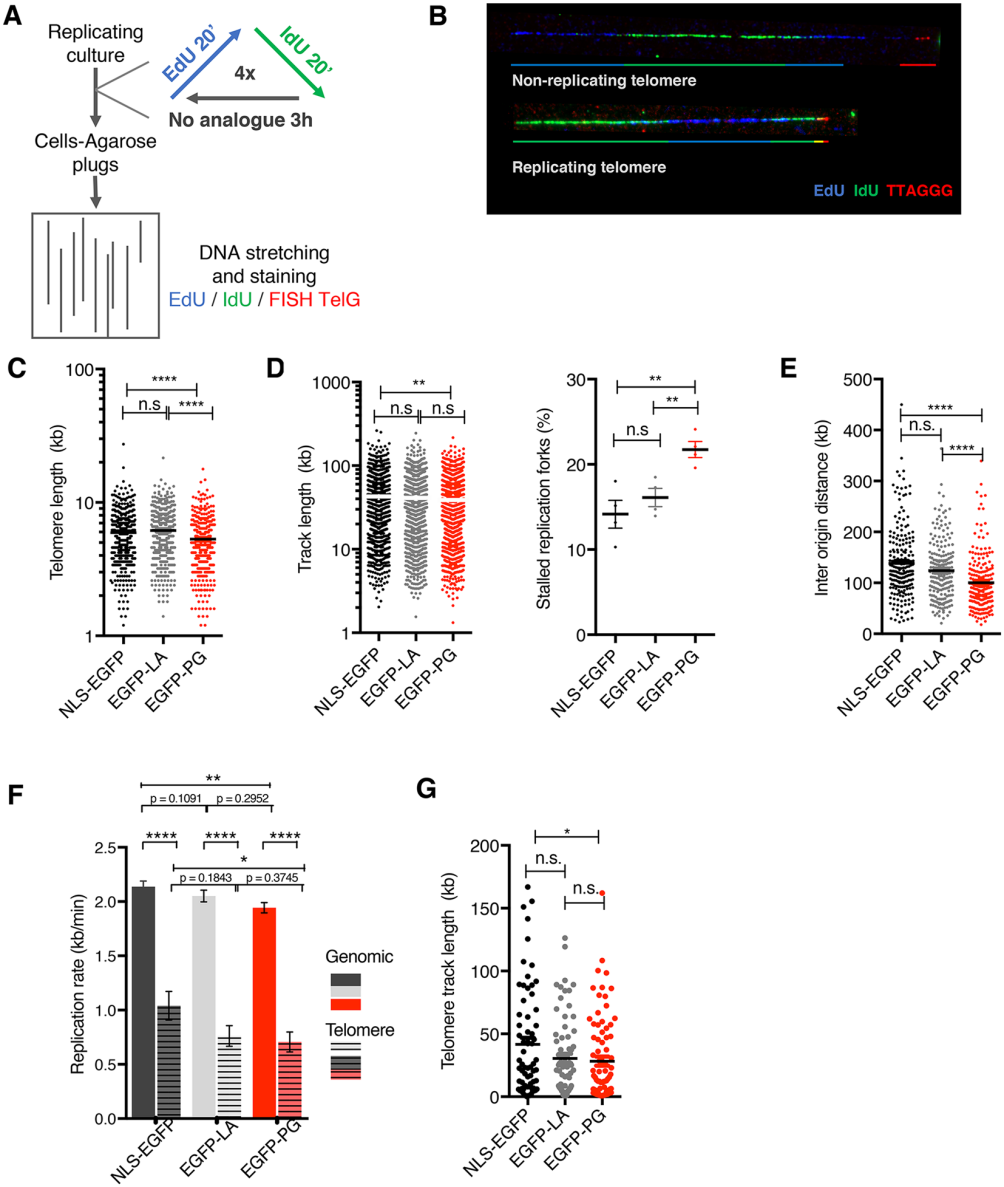
Fork progression is impaired genome-wide and at telomeres in progerin-expressing cells. (**A**) Principle of the SMARD experiment. HDF cells expressing NLS-EGFP, EGFP-LA and EGFP-PG were pulsed successively four times with the thymidine analogues EdU and IdU, followed by a chase period. Cells were collected and embedded in agarose in plugs before stretching the DNA fibers onto glass coverslips. Telomeres were visualized by FISH staining and EdU and IdU by immunofluorescence. (**B**) Examples of fibers obtained by SMARD. A non-replicating telomere corresponds to a detected FISH signal away from the EdU and IdU signals. A replicating telomere corresponds to a FISH signal located at the end of the fiber and in continuity with EdU or IdU signals. (**C**) Distribution of telomere length measured from DNA fibers performed as in (**A**). The size of the FISH signal gives an approximation of the corresponding telomere length. Mean with SEM is shown, n = 3. Statistical significance was determined using t-test (****indicates p < 0.0001). (**D,E**) Quantification of SMARD analyses at the genome-wide level in HDF cells expressing NLS-EGFP, EGFP-LA and EGFP-PG. (**D**) the graphs represent the replication track lengths (left), and the percentage of stalled replication forks in each replicate (right). (**E**) Inter Origin Distance (IOD). (**F**) Quantification of SMARD analyses at telomeres and genomic regions from HDF cells expressing NLS-EGFP, EGFP-LA and EGFP-PG. The graph represents the rate of genomic versus telomere replication. (**G**) Quantification of SMARD analyses at telomeres from HDF cells expressing NLS-EGFP, EGFP-LA and EGFP-PG. The graphs represent the telomere track length. (**D–G**) Mean with SEM is shown, n = 3. Statistical significance was determined using t-test (*indicates p < 0.05, **indicates p < 0.01, ****indicates p < 0.0001).

To further examine the impact of progerin on DNA replication, we decided to assess the progression of replication forks on DNA fibers. This technique was used in recent reports to demonstrate that progerin expression globally impaired fork progression^49^, generating a replication stress that induced a cell-intrinsic innate immune response^59^. However, how this relates to short telomeres observed in progerin-expressing cells and why telomerase expression rescued the cell growth is still unclear. The expression of fragile telomeres in both HGPS cells and progerin-expressing HDFs (Fig. 3D) suggested that the progression of forks might be affected at telomeric repeats. To test this idea, we performed **s**ingle-**m**olecule **a**nalysis of **r**eplicated **D**NA (SMARD) to measure replication progression specifically at telomeres^53,60^. Cells were pulsed successively with the thymidine analogues EdU and IdU, which are incorporated into the newly synthesized DNA, followed by a chase period. This pulse/chase cycle was repeated four times before collecting the cells for DNA stretching on glass coverslips. EdU and IdU incorporation was visualized on the stretched DNA fibers via immunofluorescence, and telomeres were labeled using telomere FISH (Fig. 5A). This experiment allows evaluation of several parameters related to the progression of the DNA replication forks, including track length, the inter-origin distance, and the proportion of stalled forks (Fig. S5A,B). Telomere fibers with no EdU/IdU incorporation were considered non-replicating (Fig. 5B). Telomere FISH on stretched DNA fibers was first used to measure telomere length^61^ in all three cell lines. Consistent with our previous data using Southern blots (see Fig. 2), telomeres were on average 1 kb shorter in EGFP-PG cells compared to NLS-EGFP and EGFP-LA control HDFs (Fig. 5C). Next, we examined replication fork progression and stability at the whole-genome level. Progerin expression in HDFs provoked a decrease in track length and replication fork rate, and an increase in fork stalling (Fig. 5D–F). As a consequence, the measured inter-origin distance (IOD) decreased in progerin cells, suggesting an activation of dormant origins to compensate for fork failures (Fig. 5E). Then, we specifically assessed replication progression at telomeres, only scoring fibers with a terminal telomere FISH signal next to EdU and/or IdU labelling (Fig. S5C). Although these experiments were technically challenging due to relatively short telomeres in untransformed HDF cells and a limited number of cells in S phase, we nevertheless managed to score enough telomere-fibers for accurate statistical analysis. We observed that replication forks were moving at a slower pace at telomeres than in other genomic regions, with rates close to 1 kb/min and 2 kb/min, respectively (Fig. 5F). This result confirmed that telomeric DNA is a difficult substrate for replication fork progression in normal cells. Progerin expression further decreased replication fork progression at telomeres with a drop to 0.7 kb/min. Consistent with a slower replication rate, we also observed shorter track length (Fig. 5G). Origins of replication were observed mostly at subtelomeric regions, and their number was not affected by progerin expression (Fig. S5D). Importantly, changes in the lamina organization by increasing the level of wild-type A-type lamins also moderately affected DNA replication. This was more obvious at telomeres, with an intermediate phenotype between EGFP-NLS control and EGFP-Pg.

### Progerin affects the replication timing signature of normal HDFs, which impairs their replication capacity

The DNA replication timing (RT) program is a highly organized and defined temporal sequence of S phase replication events. Replication timing and 3D chromatin organization are tightly connected, with early replication taking place in the nuclear interior, while chromatin at the nuclear periphery replicates late in S phase^62^. Recent work reported RT programs of a large number of fibroblasts derived from HGPS progeroid patients and healthy donors^63^. They discovered a specific RT signature of cells from HGPS donors, with regions that replicate early only in progeria but replicate later in cells from all healthy donors. This study highlighted a particular region on chromosome 3 encompassing the *TP63* gene that replicates early compared to healthy donors. In order to determine whether exogenous progerin expression could also impact HDF cells RT, we examined at the genome-wide level their spatiotemporal replication program before and after retroviral transduction of EGFP-LA and EGFP-PG. Cells were pulse-labeled with BrdU and sorted into early and late S-phase fractions by flow cytometry. Nascent BrdU‐substituted DNA of each fraction was then isolated and labeled with fluorescence dyes before hybridization on microarrays covering the human genome, with the exception of centromeric and telomeric regions, with one probe every 13 kb. The RT was then determined using the Agilent CGH algorithm. A positive log ratio value corresponds to early-replicating regions, while a negative log ratio value corresponds to late-replicating regions. We compared the RT profiles of EGFP-LA and EGFP-PG cells with the profile from non-transduced cells and found that progerin expression induced a significant change in RT for about 3% of the genome (Fig. S6A). As an example, the RT profiles of chromosome 1 are displayed Fig. S6B. Progerin expression induced a shift to earlier replication for 1.6% of chromosome 1, and a shift to later replication for only 0.2% of that chromosome. To compare our data with the one from Rivera-Mulia et al., we plotted the chromosome 3 RT profiles from LA and PG cells compared to non-transduced HDFs (Fig. S6C). Expression of progerin did shift *TP63* towards earlier replication, confirming *TP63* as a marker of HGPS as previously proposed. In conclusion, exogenous progerin expression can alter the RT signature of normal HDFs, with a tendency to hasten their replication program.

### Progerin expression limits dNTPs accessibility

**Figure 6:**
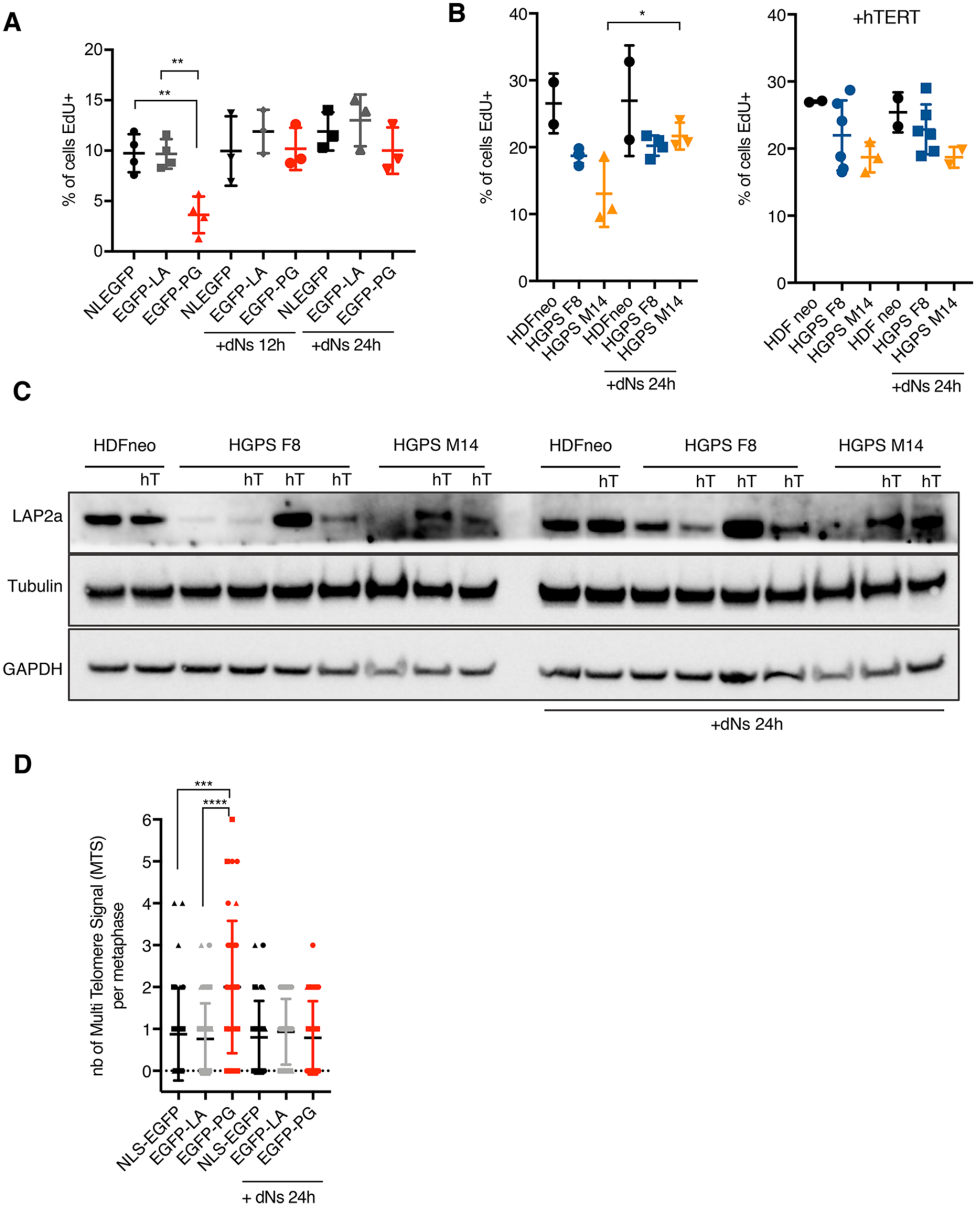
Progerin expression limits dNTPs accessibility. (**A**) Percentage of EdU positive cells in HDFs expressing NLS-EGFP (N), EGFP-LA (A), and EGFP-PG (P). When indicated, cells were supplemented with dNTPs for 12 or 24 h, then pulse-labeled with EdU for 30 min. Mean with SD is shown, n = 3. Statistical significance was determined using one-way Anova (**indicates p < 0.001). (**B**) Percentage of EdU positive cells in HDF and HGPS cells with (right) or without (left) hTERT expression. When indicated, cells were supplemented with dNTPs for 24 h, then pulse-labeled with EdU for 30 min. Mean with SD is shown, each dot corresponding to a replicate (n = 2 to n = 6 depending of the cell line). Statistical significance was determined using one-way Anova (*indicates p < 0.005). (**C**) Western blot of total extracts extracted from the indicated cell lines. When indicated, cells were supplemented with dNTPs for 24 h before collecting. (**D**) Quantification of MTS in HDF expressing the indicated constructs. The graphs represent the number of MTS per metaphase. Mean with SD is shown, n = 3. At least 30 metaphases were scored per condition in total. Statistical significance was determined using Mann Whitney test (***indicates p < 0.0005, and ****p < 0.0001).

Physiological nucleotide levels are known to be limiting for DNA synthesis. The temporal regulation of origin firing controls the number of replication forks that are active at any given time and moderates the demand for dNTPs. When the RT is not strictly respected, too many origins of replication are simultaneously activated, which in turn promotes a shortage of available dNTPs^62^. We hypothesized that a similar scenario may occur in progerin cells. Nucleotide metabolism was previously shown to be impaired in HGPS-derived cells^64^, which could further sensitize cells to a dNTP shortage. The shift to earlier RT as well as the smaller IOD measured on DNA fibers (Fig. 5E) indeed suggest that more replication forks are simultaneously active in these cells, which could limit dNTPs availability. To test this idea, HDF cells expressing NLS-EGFP, EGFP-LA and EGFP-PG were supplemented with dNTPs for 12 or 24 h, and pulse labeled with EdU for 30 min (Fig. 6A). The percentage of EdU positive cells was scored to reflect the replication capacity of the cells. As expected, progerin expression induced a twofold decrease of EdU positive cells compared to controls. Complementation of the cell medium with dNTPs was sufficient to rescue this phenotype and to restore the percentage of EdU positive cells to a level similar to controls (Fig. 6A). In patient cells, we observed that HGPS M14 cells also positively responded to dNTPs supplementation, with an increase in EdU positive cells after 24 h of treatment (Fig. 6B). The growth capacity of HGPS F8 cells was not significantly improved, but the initial percentage of EdU positive cells was much higher in this cell line. In line with this, dNTPs supplementation of HGPS cells expressing hTERT did not further increase the percentage of EdU positive cells (Fig. 6B). These cells already reached a steady replicative pace, comparable after dNTPs supplementation or hTERT expression. LAP2α expression was increased in HGPS cells after hTERT expression, as previously described^17^, and surprisingly dNTPs supplementation also impacted LAP2α levels, with an increased observed in most samples including normal HDFs (Fig. 6C). Finally, we tested whether dNTPs addition could prevent the formation of MTS during telomere replication. HDF cells expressing NLS-EGFP, EGFP-LA and EGFP-PG were grown with or without addition of dNTPs in the cell medium for 24 h, and the presence of MTS was scored on metaphase spreads. Remarkably, dNTPs supplementation completely reversed the occurrence of this phenotype (Fig. 6D). Overall, these results confirm that progerin expression alters the RT program, most likely due to changes in 3D-chromatin organization and structure, and in turn impacts replication capacity by limiting access to dNTP pools. This dNTPs shortage directly affects telomere replication, triggering the expression of fragile telomeres.

## Discussion

Overall, our work proposes a mechanistic model to describe the effects of NE dysfunction on telomere homeostasis in the context of premature aging. We propose that progerin expression alters the 3D telomere organization and their heterochromatin status. The genome-wide replication signature is perturbed, with a shortage of dNTPs that weight on telomere maintenance by inducing a telomeric replicative stress. As an outcome, telomeres shorten faster, inducing premature senescence and premature organismal aging. The strong connection between lamins, aging and telomeres we unraveled is also highly relevant for normal aging, as sporadic endogenous cryptic site leading to progerin expression has been discovered in wt aged human cells^40^.

### Progerin and telomere organization

The NE is the barrier that confines the nucleoplasm and confers, among other functions, the essential scaffold required to organize the nuclear content. Besides being tissue- and cell-type specific, the spatial organization of chromatin influences major biological processes such as regulation of gene expression, replication timing, genome stability, and senescence^21,65,66^. Using our proximity labeling technique MadID, we discovered that expression of progerin increases the interaction between telomeres and the nuclear lamina. It is unlikely that the higher level of methylation we observed is due to increased methylation accessibility of telomeric chromatin. Indeed, both wild-type LaminA and Progerin affected heterochromatin marks at telomeres (discussed below), while only progerin impacted its methylation. Whether this interaction occurs at the nuclear periphery, in the nuclear interior, or at sites of membrane invagination is still unclear. Blebbing of the NE could increase the chances of contact between lamins and chromatin. This is reminiscent of changes in nuclear architecture during senescence of human mesenchymal stem cells, where centromeres and telomeres colocalize with lamina intranuclear structures as shown by chromatin immunoprecipitation experiments^67^. Nevertheless, this enriched contact can alter telomere maintenance and perturb their dynamic properties. *Lmna*^*−/−*^ mouse cells redistribute the telomeres to the nuclear periphery^37^, however, expression of progerin most likely results in a very different outcome when it comes to chromatin dynamics. The anomalous diffusion of interphase telomeres depends on the LaminA network that creates a crosslink to restrict chromatin movements^68,69^. Therefore, progerin expression could further stabilize telomeres and limit their diffusion, potentially creating a topological stress. With these elements in mind, it is also not surprising that expression of wild-type LaminA had some consequences on chromatin marks at telomeres. LaminA can occupy both heterochromatin and euchromatin domains^70^. An increase in LaminA will create an unbalanced A- and B-Type organization within the nuclear lamina, perturb the pool of nucleoplasmic LaminA^71^, and alter chromatin state.

### Accurate replication timing is crucial for genome maintenance

Alteration of heterochromatin at the genome level has been linked to changes in the replication timing signature in HGPS^63^. In our setting, a significant proportion of the genome replicated earlier in progerin cells compared to controls. The changes were mild, but we analyzed the profiles of cells 2 weeks post selection after EGFP-PG transduction, to collect exponentially growing cultures. Changes would probably increase after longer periods of progerin expression as it happens in HGPS patient cells. We did not specifically determine the replication timing of telomeres, which are known to replicate throughout S phase in normal human cells. While heterochromatin marks were decreased at telomeres, which could favor a shift to earlier replication, their stronger interaction with the nuclear lamina could in contrary favor a delay. Nevertheless, the shift toward earlier replication observed genome-wide will limit the levels of “building blocks”, i.e. dNTPs, that can in turn affect any active replication fork. This is what we observed at telomeres, with a supplementation of dNTPs sufficient to alleviate the replication stress induced by progerin expression. This result is consistent with the finding that HGPS cells have an altered de novo purine synthesis machinery with a downregulation of ribose-phosphate pyrophosphokinase 1 (PRPS1), essential for nucleotide synthesis^64^.

### Replication stress, lamins and telomeres

Compared to the global genome, our SMARD analysis revealed that forks progress slower at telomeres, a region known to be difficult to replicate^53^. Progerin expression further impaired the fork progression, consistent with the fragile telomere we observed on metaphase spreads. Recent work suggest that heterochromatin loss is a determinant of progerin-induced DNA damage that occurs in late S phase^72^. Our work reveal a similar behaviour at telomeres, with a diminution in H3K9 methylation and replication defects that promotes faster telomere shortening. Other mechanisms could also promote replication stress in progerin cells. Several lines of evidence link nuclear A- and B-Type lamins to DNA replication aptitude, via interaction with sites of DNA replication, and/or PCNA^49,73–76^. These findings also explain why expression of wild-type LaminA mildly influenced fork progression. The protein AKT-interacting protein (AKTIP) could also be at play. AKTIP has been reported to preferentially localize at the nuclear periphery, but also to interact with shelterin proteins and be required for normal telomere replication^77,78^. Since AKTIP is depleted in HGPS cells^78^, its implication should be further assessed. Lastly, although we could not detect a change in shelterin expression in total extracts, and no obvious change in TRF1 and TRF2 binding to telomeric repeats using ChIP-dot blot (Fig. 3B), ChIP-Q-PCR experiments revealed a small decrease in TRF1 binding to telomeres after progerin expression (Fig. 1I). Since TRF1 promotes efficient telomere replication^53^, it would be interesting to further assess the role of TRF1 in progerin-induced fragile telomeres.

### Relevance of telomere defects for HGPS organismal premature aging

HGPS patients appear normal at birth, but develop severe abnormalities within the first two-years of life. As telomere dysfunction stands out to be a key element in the fitness of progerin cells, long telomeres at birth might delay the clinical signs of the disease in patients. Even if only two patient cell lines were studied here, all phenotypes appeared more pronounced in HGPS M14 compared to F8, which correlates with patient age and initial telomere length. Similarly, up to 5 PDs were necessary to observe cell growth delays after ectopic expression of progerin in normal cells. These observations suggest that telomere length is determinant for the onset of HGPS-related phenotypes, such as premature senescence. It could explain the discrepancy in the literature related to the severity of the phenotypes induced by progerin expression, notably on the extent of DNA damage or onset of growth delay^45,79^.

## Method

### Plasmids

pBabe-EGFP-LaminA and pBabe-EGFP-Progerin were from Tom Misteli (Addgene plasmids #17662 and #17663)^80^. pBabe-EGFP-LaminA express the LaminA precursor (pre-LaminA), corresponding to a protein of 665 amino acids. Pre-LaminA is then processed into the mature LaminA, a protein of 646 amino acids. pBabe-EGFP-Progerin express the trunctated form of LaminA found in HGPS cells, a protein of 615 amino acids. pLPC-NLS-EGFP: NLS-EGFP sequence was cloned into pLPC vector by PCR using primers EcoRI-NLS-EGFP-For and XhoI-EGFP-Rev^34^. pLPC-hTERT and HPV16 E6 and E7 vectors were obtained from Jan Karlseder (Salk Institute, CA, USA).

### Cell lines and culture

Normal Human Dermal Fibroblasts (HDF) derived from adults or neonatal tissues were from Sigma. HGPS dermal fibroblasts were from Coriell Cell Repository (NIA Aging Cell Culture Repository): AG06297 (8 years old male—M8), AG11498 (14 years old male—M14), AG11513 (8 years old female—F8). All cell lines were cultured in Glutamax-DMEM medium (Gibco) supplemented with 15% (v/v) fetal bovine serum (Gibco) and non-essential amino acids (Gibco), at 37 °C with 7.5% CO_2_ and 5% O_2_. Retroviruses were produced and cells were transduced as described^81^. To generate growth curves, cells were passaged and expanded until their growth plateau. Population doublings (PD) were determined using the formula: PD = log_2_(C2/C1), where C2 is the number of harvested cells, and C1 the number of seeded cells. All other experiments were performed in freshly transduced cells, i.e. 2 to 4 weeks post-selection, to assess progerin-related phenotypes independently on senescence entry.

### Cell synchronization

For cell synchronization, cells were grown to 50% confluence and incubated for 17 h with 2 mM of thymidine (Sigma). Cells were washed three times with PBS, and then were supplemented with fresh medium containing 10 µmol 2-deoxycytidine (Sigma) to facilitate their release. A double thymidine block was performed when indicated.

### Western blotting

Whole protein extracts were obtained by treating cell pellets with LDS lysis buffer NuPAGE (Thermo Fisher), heated at 95 °C for 10 min. The equivalent of 10^5^ cells was loaded and resolved on pre-cast 4–12% SDS-PAGE gradient gels (Invitrogen) for 45 min–1 h at 100 V. Transfer of protein samples from SDS-PAGE gels to nitrocellulose was performed with Towbin transfer buffer (25 mM Tris Base, 192 mM glycine, 20% methanol) for 1 h at 100 V. Membranes were overlaid with western blotting substrate for 5 min (Clarity™, BioRad) before visualization with a ChemiDoc™ Imaging System (BioRad). Original blots are displayed in Figs. S7 and S8.

### Immunofluorescence

Human fibroblasts were grown to 50% confluence on glass coverslips (1.5 thickness) and fixed with 4% paraformaldehyde for 10 min at RT. After three washes with PBS, cells were permeabilized with 0.5% Triton X-100 in PBS and incubated with a blocking solution (0.2% (w/v) cold water fish gelatin, 0.5% (w/v) Bovine Serum Albumin (BSA) in 1× PBS) for 30 min at RT. Incubation of primary antibodies was performed for 2 h at RT or overnight at 4 °C. Alexa488/546/647-conjugated secondary antibodies (Invitrogen) were incubated for 45 min at RT. For the detection of Ultrafine Anaphase Bridges (UFBs), the procedure described in Bizard et al., was followed^54^. Coverslips were mounted with Mowiol mounting medium (24% w/v Glycerol, 9.6% w/v Mowiol 4–88, 0.1 M Tris–HCl pH 8.5, 2.5% w/v Dabco). Cells were imaged with an Olympus BX63 epifluorescence microscope or ZEISS Airyscan confocal microscope. Image analysis was carried out using ImageJ and Cell Profiler.

### Telomere FISH on metaphase chromosomes

Human fibroblasts were grown to ~ 40% confluence and incubated with 0.1 μg/ml colcemide for 4 h. Cells were harvested by trypsinization, swollen in 0.075 M KCl for 7 min at 37 °C, and fixed in methanol/acetic acid (3:1) overnight at 4 °C. Fixed cells were spread on water-wetted microscope glass slides, washed with fresh fixative, and dried on a humidified 80 °C heat block. After aging overnight, slides were washed in 1× PBS for 5 min followed by consecutive incubation with 75%, 95% and 100% ethanol. Slides were allowed to air dry for 30 min before applying hybridizing solution (70% formamide, 1 mg/ml blocking reagent (Roche), 10 mM Tris–HCl pH 7.2) containing diluted 1:250 FITC-OO-(CCCTAA)3 PNA probe (Panagene Cat. No. F1009). Spreads were denatured for 3 min at 80 °C on a heat block and hybridized at RT for 2 h. Slides were washed twice with 70% formamide, 10 mM Tris–HCl (15 min each wash), followed by three washes in 0.1 M Tris–HCl, pH 7.0, 0.15 M NaCl, 0.08% Tween-20 (5 min each). Chromosomal DNA was counterstained with DAPI added to the second wash. Slides were mounted with Mowiol mounting medium and metaphase chromosomes were imaged using an Olympus BX63 microscope. Images were processed using ImageJ software.

### Cell fractionation and co-immunoprecipitation

Cell pellets from 5 to 10 × 10^6^ cells were collected swollen for 15 min on ice in swelling buffer (10 mM Hepes pH 7.8, 10 mM KCl, 2 mM MgCl_2_, 0.1 mM EDTA, 1 mM DTT) supplemented with protease inhibitors (Roche). After addition of 20 μl of 10% NP40, cells were briefly vortexed and centrifuged for 30 s at maximum speed at 4 °C. The supernatant (cytoplasmic fraction) was collected. The cell pellet was resuspended with 50 μl of lysis buffer (50 mM Hepes pH 7.8, 50 mM KCl, 300 mM NaCl, 0.1 mM EDTA, 10% Glycerol, 1 mM DTT) supplemented with protease inhibitors (Roche), and vortexed for 20 min at 4 °C. Nuclear lysates were centrifuged at maximum speed for 15 min at 4 °C to remove the cell debris. The supernatant corresponding to nuclear fraction was used to perform the co-immunoprecipitation with the mMACS GFP Epitope Tag Isolation kit (Miltenyi Biotec) according to manufacturer’s instructions.

### Senescence-associated-β-galactosidase assay

Fibroblasts seeded on 2-well Ibidi^®^ slides were stained with SA‐β‐Gal following manufacturer’s instructions (Senescent Cell Histochemical Staining Kit, CS0030-1KT, Sigma‐Aldrich) for 12 h to 15 h. Blue‐stained cells expressing β‐galactosidase (senescent cells) were observed 4× objective under bright‐field microscopy.

### BrdU labeling and flow cytometry

Cells were pulse labeled with 50 μM BrdU for 90 min prior to harvesting. Cells were fixed with ice cold 70% ethanol and stored at 4 °C overnight or longer. S-phase cells were detected using the FITC Mouse Anti-BrdU Set (Becton Dickinson) following the manufacturer’s instructions.

DNA content was analyzed by PI. Briefly, cells were resuspended in 0.5 ml PBS/2 mM EDTA containing 50 μl 1 mg/ml propidium iodide (PI) and 20 μl 10 mg/ml RNase A and incubated at 37 °C for 30 min. Cells were subjected to FACS analysis using a CytoFLEX S flow cytometer (Beckman Coulter). A minimum of 10,000 cells per samples were gated. The percentage of BrdU-positive cells and the distribution of cells in the different phases of the cell cycle based on PI content were analyzed with the FlowJo software. The standard gating strategy included FSC/SSC for cell morphology, FSC-A/FSC-H for single cells, FL1-A::AF488-A (Alexa 488 for BrdU)/ FL8-A::IP-A (Propidium Iodide) were used. After using scatter gates and cell doublet exclusion, analytical cell cycle analysis was performed based on BrdU and Propidium iodide incorporation as a function of DNA content.

### Replication timing profiles

Exponentially growing HDF cells non-transduced or expressing NLS-EGFP, EGFP-LA and EGFP-PG were pulse-labeled with 50 μM BrdU for 90 min, washed three times in PBS, and then a minimum of 10 million cells per sample were fixed in 70% ethanol and stored at − 20 °C. Fixed samples were resuspended in PBS-RNAseA (0.5 mg/ml) and 50 μg/ml propidium iodide, incubated for 30 min at RT prior to cell sorting. 100,000 cells were sorted in two fractions, S1 and S2 that correspond to early and late fractions respectively, using the INFLUX 500 or the Astrios cell sorter. Both S1 and S2 fractions were treated with 0.2 mg/ml proteinase K in lysis buffer (50 mM Tris pH 8, 10 mM EDTA, 300 mM NaCl, 0.5% SDS) for 2 h at 65 °C in the dark or overnight. DNA was extracted and sonicated to obtain fragments of about 500–1000 bp. DNA was then denatured at 95 °C for 5 min and snap cooled for 10 min. Nascent DNA was immunoprecipitated using the IP-STAR apparatus with the indirect method option (Diagenode) using 10 μg of anti-BrdU antibodies (BD Biosciences, #347,580), and purified by phenol–chloroform. The quality of the enrichment of S1 and S2 fractions was verified by qPCR using primers specific to regions replicating early or late. Next, whole genome amplification was performed using the WGA amplification kit (Sigma) to obtain at least 500 ng of DNA. After amplification, S1 and S2 were labeled with Cy3 and Cy5 ULS molecules (Genomic DNA ULS labeling Kit, Agilent) according to the manufacturer’s protocol. The samples were hybridized on 4 × 180 K human microarrays (Agilent, genome reference hg18) that covers the whole genome with one probe every 13 kb (11 kb in RefSeq sequences) following the manufacturer’s protocol. Microarrays were scanned with an Agilent’s Hi-Resolution C Scanner with a resolution of 3 μm and the autofocus option. Replication timing data were analyzed using the *limma* R package as in Ref.^82^. The loess method was used to normalize within arrays, and the quantile method was used to normalize in between arrays. The normalized profiles of each chromosome were then smoothed using loess regression fitting with a bandwidth of 3 Mb. We combined the smoothed profiles of two replicates to obtain the average RT profile of each chromosome in each condition. The 95% confidence interval (CI) obtained from the loess regression fitting of the average profiles using the *loess.ci* function of the *spatialEco* R package, and the non-transduced condition was used as reference. Regions where the CI of the condition of interest (e.g. EGFP-LA or EGFP-PG) does not intersect with the CI of the non-transduced condition correspond to regions that replicate later (lower RT values in condition of interest) or earlier (greater RT values) than the non-transduced condition. Raw data are available on Mendeley: 10.17632/352fgv8x99.1#folder-e69477f7-d849-4889-baa4-00749cfdda38

### Chromatin immunoprecipitation

Cells were fixed with 1% formaldehyde in PBS for 20 min at RT, washed with 1× PBS, and lysed in 1% SDS, 50 mM Tris–HCl, pH 8.0, 10 mM EDTA at a density of 10^7^ cells/ml. DNA was sheared into chromatin fragments < 1 kb using a Bioruptor Plus sonicator (Diagenode). After clearing of the lysates, 0.5-1 mg of protein was used per IP. Samples were diluted 10X with dilution buffer (0.01% SDS, 1.1% Triton X-100, 1.2 mM EDTA, 16.7 mM Tris–HCl, pH 8.0, and 150 mM NaCl) before addition of the indicated antibodies and incubation on a wheel overnight at 4 °C. Immunoprecipitates were pulled down with Protein-A/G coated magnetic beads (Invitrogen), and washed with 0.1% SDS, 1% Triton X-100, 2 mM EDTA, pH 8.0, 20 mM Tris–HCl, pH 8.0, containing 150 mM NaCl for the first two washes, and 500 mM NaCl for to additional washes. A final wash was performed with TE buffer. IP chromatin was eluted from the beads with 500 μl 1% SDS, 50 mM Tris–HCl pH8, 10 mM EDTA pH8. After addition of 20 μl of 5 M NaCl, crosslinks were reversed overnight at 65 °C. Samples were supplemented with 20 μg DNase-free RNase, and incubated at 37 °C for 30 min. DNA was extracted by phenol–chloroform and precipitated overnight at − 20 °C. *For dot blots* IP samples were then denatured at 95 °C for 5 min, snap-cooled on ice and dot blotted onto N + Hybond membranes in 2× SSC. Membranes were denatured with 1.5 M NaCl, 0.5 N NaOH for 10 min, then neutralized with 1 M NaCl, 0.5 M Tris–HCl, pH 7.0 for 10 min before crosslinking using a Stratalinker UV crosslinker (Stratagene) at 70,000 µJ/cm^2^. Telomeric probes 5′-(TTAGGG)_4_-3′ and centromeric probes 5′-GTTTTGAAACACTCTTTTTGTAGAATCTGC-3′ were radiolabeled by incubating 100 pmol of each with 50 μCi of ^32^P-ATP and 50 units of T4 Polynucleotide Kinase (Thermo Fisher Scientific) in 50 μl of 1× PNK buffer A for 1 h at 37 °C. The reactions were heat inactivated for 10 min at 75 °C and purified using the Micro Bio-Spin P-30 (Bio-Rad). The membranes were incubated for 30 min at 65 °C in Church Mix hybridization buffer (500 mM NaPi pH 7.2, 1 mM EDTA pH 8.0, 7% SDS, 1% BSA), and then overnight at 65 °C with 10 ml of hybridization buffer containing the radiolabeled probe. The membranes were washed 4 times in 2× SSC at 65 °C before exposure to a PhosphorImager screen. Signals were visualized with a Typhoon phosphorimager and quantified with ImageJ software. *For qPCR* IP samples were resuspended in 30 μl of TE buffer. Telomeric DNA was detected using primers as described in Ref.^43^.

### Southern blots of terminal restriction fragments (TRFs)

Analysis of telomere length was performed as in Ref.^83^. Quantification of the signal was done using the ImageJ software.

### Genomic DNA and telomere purification for MadID

Genomic DNA was isolated using the Qiagen Blood & Cell Culture DNA Midi Kit (Genomic-tip 100/G) according to the manufacturer’s recommendations together with RNAse treatment (200 µg/ml of RNAseA (Sigma) and RNase Cocktail™ Enzyme Mix (Ambion) (2.5 U/ml RnaseA; 100 U/ml RnaseT1) at 37 °C for 1 h). Telomere isolation was based on a published method with some modification^84^. Double stranded genomic DNA (50 µg) was digested overnight with AluI, HinfI, HphI and MnlI (0.5 U/mg) restriction enzymes in 300 ml reaction volume to release intact telomeric fragments. Reactions were adjusted to 1× SCC and 0.1% Triton X-100, and the digested DNA was then annealed with a biotinylated oligonucleotide (Bio-5′-ACTCC(CCCTAA)_3_-3′) (3.5 pmol) by controlled stepwise cooling from 80 to 25 °C (1 °C/min) using a thermocycler. Then 5% of samples was collected as an input and streptavidin-coated magnetic beads (18 µl, Invitrogen, M-280) prewashed with 1× PBST and blocked for 1 h with 5× Denhardt solution (0.1% Ficoll (type 400), 0.1% polyvinylpyrrolidone, and 0.1% bovine serum albumin), were incubated with the annealed samples overnight in a rotator end-over-end at 6 r.p.m. and 4 °C. Beads were collected against the side of the tubes by applying a magnet (Invitrogen), and the unbound fraction was collected. The beads were washed four times with 1× sodium chloride–sodium citrate (SSC), 0.1% Triton X-100, and once with 0.2× SSC. Beads were resuspended in 50 µl elution buffer and telomeres were slowly eluted by heating the tubes at 50 °C for 20 min. The elution was repeated with 50 µl of elution buffer. The quality of telomere purification was assessed by qPCR. Telomeric DNA was detected using primers as described in Ref.^43^. The presence of genomic DNA was assessed using primers directed against a single genomic locus as in Ref.^43^. Standard curves were performed on genomic DNA or on 800 bp of telomeric DNA purified from a plasmid to validate the primer pairs. To assess the impact of m6A methylation on PCR efficiency, the 800 bp telomeric fragment was in vitro methylated with recombinant M.EcoGII (NEB). Briefly, DNA was methylated in 50 μl of 1× dam Methyltransferase Reaction Buffer (50 mM Tris–HCl pH 7.5, 5 mM β-ME, 10 mM EDTA) supplemented with 80 µM S-adenosylmethionine (SAM) for 2 h at 37 °C. Fold enrichment were calculated over the INPUT corresponding to total genomic DNA.

### Immunodot blot detection of m6A

Immunodot blots of purified telomeric DNA and INPUTs or Lambda DNA were performed using the BioRad 96-well Bio-Dot® apparatus. Positively charged Amersham Hybond-N+ membranes (GE Healthcare) and Whatman filter papers (GE Healthcare) preincubated with 2× SSC buffer were assembled onto the apparatus. Heat-denatured (98 °C, 10 min; on ice, 5 min) DNA samples were loaded on the membrane via vacuum blotting, then the wells were washed with 2× SSC. The membrane was denatured and neutralized sequentially by placing it on top of a Whatmann filter paper (DNA face up) saturated with denaturing solution (1.5 M NaCl, 0.5 M NaOH) for 10 min at RT and neutralization solution (0.5 M Tris–HCl, pH 7.0, 3 M NaCl) for 10 min at RT. The membrane was crosslinked with UV at 70,000 µJ/cm^2^ and blocked for 1 h in 5% nonfat dry milk and 0.1% TBST (0.1% Tween-20 in 1xTBS, pH7.4). Subsequently, m6A antibody (Synaptic Systems) was diluted to 1:2000 in 5% nonfat dry milk and 0.1% TBST, and incubated overnight at 4 °C. Following 3 washes with 0.1% TBST, a HRP-conjugated secondary antibody was applied for 45 min at room temperature. After 3 washes with 0.1% TBST, the chemiluminescence signal was visualized using ChemiDoc™ Imaging System (BioRad). The signal was quantified using the ImageJ software. The intensity of the m6A signal was normalized to the amount of telomeric DNA purified in each sample measured by qPCR.

### EdU incorporation, QIBC and DNA damage labeling

For 53BP1 and yH2AX immunostaining, HDFs cells expressing NLS-EGFP, EGFP-LA and EGFP-PG were seeded on coverslips (1.5 thickness; 13 mm of diameter). When indicated, cells were treated with 0.2 μM aphidicolin (Sigma) for 24 h and 10 μM of EdU (Invitrogen C10340) was added for 30 min before fixation in 4% PFA/1X PBS. Cells were then permealized with 0.5% Triton X-100 in 1× PBS and incubated with a blocking solution (0.2% (w/v) cold water fish gelatin, 0.5% (w/v) bovine serum albumin (BSA) in 1X PBS) for 30 min at RT. Detection of EdU was performed prior to incubation with the primary antibodies using the Click-iT™ Plus EdU Alexa Fluor™ 647 Imaging Kit according to the manufacturer’s instructions (Thermo-Fisher Scientific). 53BP1 primary antibody (Santa Cruz sc-22760—1/200) or yH2AX (Biolegend 613402—1/1000) were used for immunofluorescence following the above protocol. Image acquisition of multiple random fields was carried out on a wide field DM6000 Leica microscope equipped with a 40 × dry objective using a Qimaging Retiga R6 camera driven by Metamorphe software. 10 to 20 images per condition were acquired randomly and processed for automated analysis with the Cell Profiler 2.1.1. image analysis software. DAPI signal was used for segmentation of the nuclei according to intensity threshold, generating a mask that identified each individual nucleus as an individual object. This mask was applied to quantify pixel intensities in the different channels for each individual cell/object. The values quantified for EdU and DAPI staining per cell were graph plotted by dual-parameter (EdU *vs* DNA) generating diagrams in a flow-cytometry-like fashion (QIBC Quantitative-based image Cytometry) for each cell condition. This approach allows the assignment of cells to G1, S or G2/M phases. Images were assembled with ImageJ software.

### Nucleoside supplementation

Embryomax^®^ Nucleosides 100× (Merck, ES-008-D) were added to cell culture media at a final concentration of 1× for 12 or 24 h as indicated in the figures. EdU incorporation and detection were performed as described in the previous section.

### SMARD analysis

Single-molecule analysis of replicated DNA was performed as described previously^53,60^. In brief, HDF cells cultured at 70–80% confluence were sequentially labeled four times with 100 µM IdU (20 min), 15 µM EdU (20 min), and analogue-free media for 3 h. Next, cells were harvested by trypsinization, embedded in 2% low melting agarose plugs (1.3 × 10^6^ cells per plug) and subjected to proteinase K digestion at 50 °C overnight (200 μl 0.5 M EDTA pH 8.0, 25 μl 10% (w/v) Sarcosyl/0.5 M EDTA, 50 μl 20 mg/ml Proteinase K). Molecular combing was performed using the Molecular Combing System (Genomic Vision S.A.) with a constant stretch factor of 2 kb/μM using Molecular Combing coverslips vinyl silane coverslips (20 × 20 mm; Genomic Vision S.A.), according to the manufacturer's protocol. After combing, coverslips were dried for 4 h at 60 °C. The quality and integrity of combed DNA fibers were verified with the YoYo‐1 DNA counterstain (Molecular Probes). Coverslips were denatured for 15 min in alkali‐denaturing buffer (0.1 M NaOH, 0.1% β‐mercaptoethanol in 70% ethanol) and fixed by addition of 0.5% glutaraldehyde for 5 min. Replication tracks were visualized by detection of halogenated nucleotides using anti‐IdU and anti‐EdU antibodies. For the replication fork progression analysis at telomeric regions, telomeric DNA was visualized by hybridization with an TAMRA–OO‐(CCCTAA)_3_ PNA probe and replication tracks co‐localized with PNA staining were scored. Track length was determined using the length of the IdU track (second pulse). Stalled forks are seen as labelled during the first pulse of EdU, but not IdU signal was detected.

### Statistical analysis

GraphPad Prism version 7.0c for Mac was used for statistical analysis.

### Drugs used in this study

**Table:** 

EdU	Invitrogen C10340
BrdU	SIGMA 19-160
Aphidicolin	SIGMA A-0781
Bleomycin	SIGMA B-8416
Thymidine	SIGMA T1895
Embryomax^®^ Nucleosides 100×	Merck, ES-008-D

### Antibodies used in this study

**Table:** 

TRF1	HOME MADE ab129177 Abcam rabbit mono ab10579 Abcam mouse mono
TRF2	HOME MADE ab13579 Abcam mouse mono
Lamin B	sc-6216 Santa Cruz goat poly
SUN1	HPA008346 Sigma rabbit poly
LaminA/C	sc-7292 Santa Cruz mouse mono
H3K9me2	ab1220 Abcam mouse mono
H3K9me3	ab8898 Abcam rabbit poly
H3	ab1791 Abcam rabbit poly
H2AX-phospho (Ser139)	613402 Biolegend mouse mono ab2893 Abcam rabbit poly 05-636 Millipore mouse
53BP1	sc-22760 Santa Cruz rabbit poly
PICH	04-1540 Millipore mouse mono
beta-Actin	sc-69879 Santa Cruz mouse mono
LAP2alpha	SAB4200238 SIGMA mouse mono
V5	13202 Cell signaling, rabbit poly
GFP-HRP	130-091-833 Miltenyi
m6Adenine	202003 SYSY rabbit poly
POT1	ab47082 Abcam rabbit poly
TPP1	ab57595 Abcam mouse mono

## Supplementary Information

Supplementary Information.

## Data Availability

Raw replication timing data are available on Mendeley: 10.17632/352fgv8x99.1#folder-e69477f7-d849-4889-baa4-00749cfdda38. All other relevant data supporting the key findings of this study are available within the article and its Supplementary Information files or from the corresponding authors upon reasonable request.
