# Supplementary material for: Progerin impairs 3D genome organization and induces fragile telomeres by limiting the dNTP pools
Source: Sci Rep. 2021 Jun 23;11:13195. doi: 10.1038/s41598-021-92631-z (PMC8222272; doi:10.1038/s41598-021-92631-z)

Progerin impairs 3D genome organization and induces fragile telomeres by limiting the dNTP pools

Anna Kychygina[1,4], Marina Dall'Osto[1], Joshua A. M. Allen[2], Jean-Charles Cadoret[3], Vincent Piras[1], Hilda A. Pickett[2], Laure Crabbe[1*]

Affiliation:
[1]Molecular, Cellular and Developmental biology department (MCD), Centre de Biologie Integrative (CBI), University of Toulouse, CNRS, UPS, 31062 Toulouse, France
[2]Telomere Length Regulation Unit, Children's Medical Research Institute, Faculty of Medicine and Health, University of Sydney, Westmead, NSW 2145, Australia.
[3]Université de Paris, CNRS, Institut Jacques-Monod, UMR7592, France.
[4]Toulouse Institute for Infectious and Inflammatory Diseases (Infinity), INSERM UMR1291, CNRS UMR5051, UT3, 31059 Toulouse, France

[*]Corresponding author: laure.crabbe@univ-tlse3.fr

https://orcid.org/0000-0001-7764-3600

Contents
Supplementary Figures 1-6 and Legends

SUP. FIGURE 1

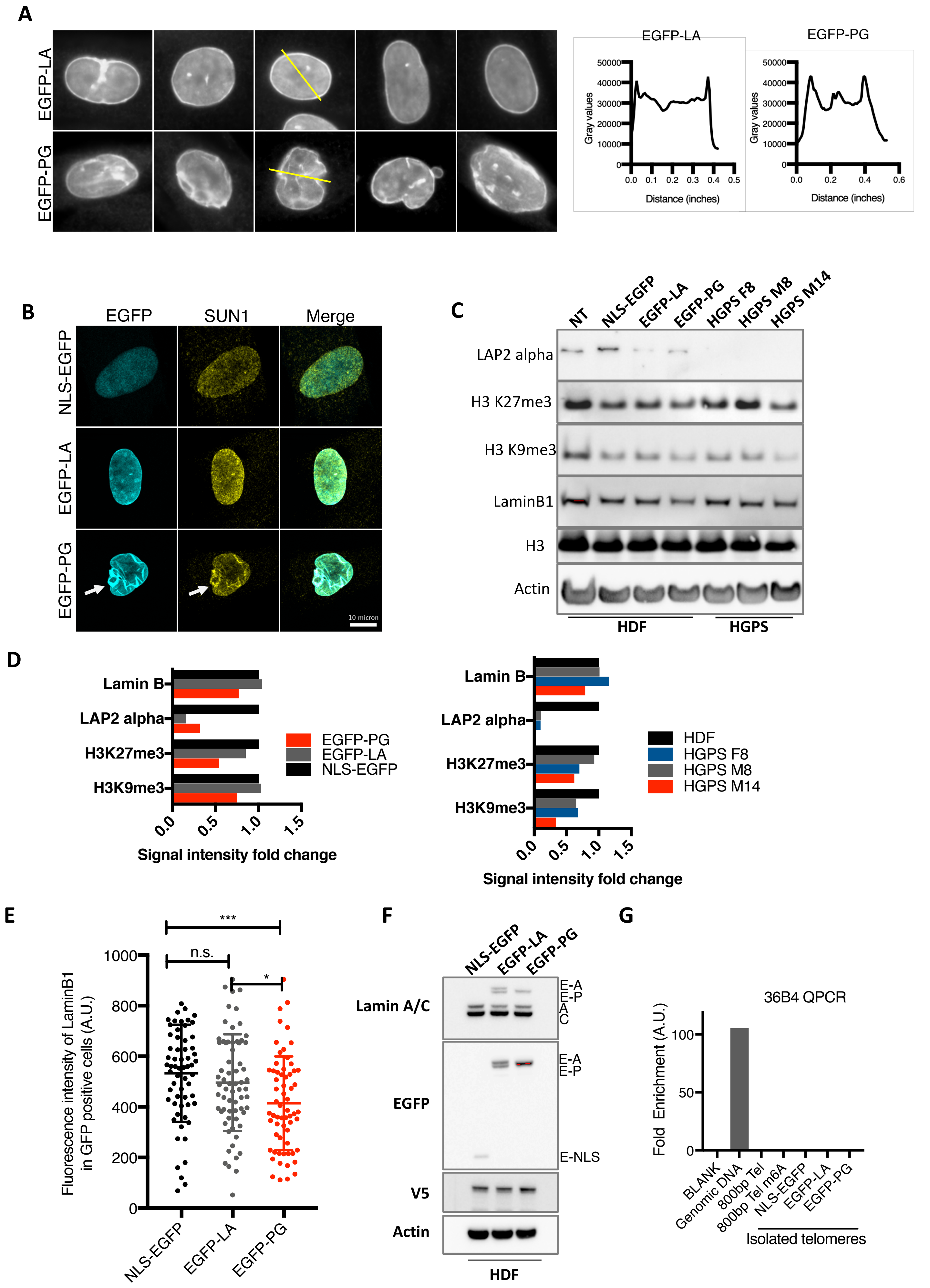
A
EGFP-LA
EGFP-PG
Gray values
Distance (inches)
B
EGFP
SUN1
Merge
NLS-EGFP
EGFP-LA
EGFP-PG
10 micron
C
NT
NLS-EGFP
EGFP-LA
EGFP-PG
HGPS F8
HGPS M8
HGPS M14
LAP2 alpha
H3 K27me3
H3 K9me3
LaminB1
H3
Actin
HDF
HGPS
D
Lamin B
LAP2 alpha
H3K27me3
H3K9me3
Signal intensity fold change
EGFP-PG
EGFP-LA
NLS-EGFP
HDF
HGPS F8
HGPS M8
HGPS M14
E
Fluorescence intensity of LaminB1 in GFP positive cells (A.U.)
***
n.s.
*
NLS-EGFP
EGFP-LA
EGFP-PG
F
NLS-EGFP
EGFP-LA
EGFP-PG
Lamin A/C
E-A
E-P
A
C
EGFP
E-NLS
V5
Actin
HDF
G
36B4 QPCR
Fold Enrichment (A.U.)
BLANK
Genomic DNA
800bp Tel
800bp Tel m6A
NLS-EGFP
EGFP-LA
EGFP-PG
Isolated telomeres

Supplementary Figure 1. Related to Figure 1

A- Five representative confocal images (middle stack) of cells expressing EGFP-LA or EGFP-PG. Quantification of the grey values over the distance (yellow lines) is shown on the right. B- Representative immunostaining of HDF cells expressing the indicated constructs. EGFP (cyan), SUN1 (yellow), and merge. The arrows point to a region at the NE with accumulation of progerin and Sun1. Scale bar: 10mm. C- Western blots of whole cell extracts of the indicated cell lines. D- Quantification of (B). The graphs represent the signal intensity fold change using NLS-EGFP (left) or HDF (right) as a reference. E- Quantification of LaminB1 fluorescence intensity in HDF expressing NLS-EGFP, EGFP-LA, or EGFP-PG. Statistical significance was determined using Mann Whitney test (* indicates $p<0.05$, and *** $p<0.0005$). At least 60 nuclei were scored per cell line. F- Western blots of whole cell extracts of the indicated cell lines. The EGFP antibody detects bands corresponding to NLS-EGFP (E-NLS), EGFP-LA and EGFP-PG (E-A/E-P). The EGFP-LA sample presents two bands, most likely corresponding to pre-LaminA (expressed) and mature LaminA form after *in vivo* processing. The V5 antibody detect the M.EcoGII-LaminB construct. G- qPCR analyses of MadID using 36B4 primers to amplify a single genomic locus. The following templates were used: genomic DNA, a fragment of 800bp of telomeric repeats before or after *in vitro* methylation with M.EcoGII, and isolated telomeres from HDF expressing NLS-EGFP, EGFP-LA, or EGFP-PG as indicated.

SUP. FIGURE 2

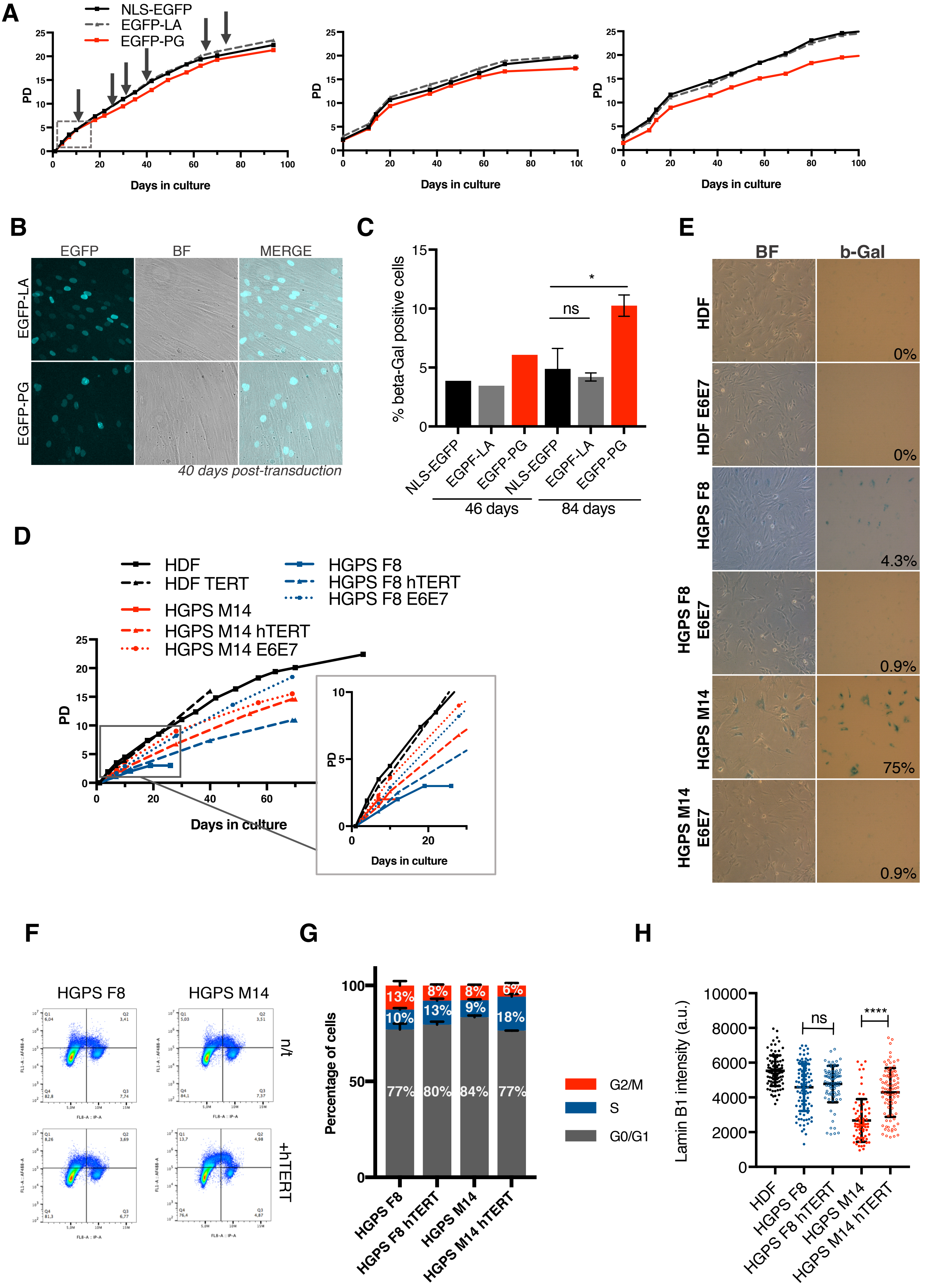
A
NLS-EGFP
EGFP-LA
EGFP-PG
PD
Days in culture
B
EGFP
BF
MERGE
EGFP-LA
EGFP-PG
40 days post-transduction
C
% beta-Gal positive cells
ns
*
NLS-EGFP
EGFP-LA
EGFP-PG
46 days
84 days
E
BF
b-Gal
HDF
HDF E6E7
HGPS F8
HGPS F8 E6E7
HGPS M14
HGPS M14 E6E7
0%
0%
4.3%
0.9%
75%
0.9%
D
HDF
HDF TERT
HGPS M14
HGPS M14 hTERT
HGPS M14 E6E7
HGPS F8
HGPS F8 hTERT
HGPS F8 E6E7
PD
Days in culture
F
HGPS F8
HGPS M14
n/t
+hTERT
G
Percentage of cells
13%
10%
77%
8%
13%
80%
8%
9%
84%
6%
18%
77%
G2/M
S
G0/G1
HGPS F8
HGPS F8 hTERT
HGPS M14
HGPS M14 hTERT
H
Lamin B1 intensity (a.u.)
ns
****
HDF
HGPS F8
HGPS F8 hTERT
HGPS M14
HGPS M14 hTERT

Supplementary Figure 2. Related to Figure 2

A- Examples of growth curves of HDF expressing NLS-EGFP, EGFP-LA, or EGFP-PG. Each graph represents the cumulative population doublings (PD) as a function of days of growth. The arrows on the top graph point to the time of collection of cells for southern blots analyses shown in Fig. 2A. The dash square on the first graph highlights the first PDs were no difference in cell growth is observed between the different cell lines. B- Live HDF cells expressing EGFP-LA or EGFP-PG, 40 days post-transduction. The EGFP signal (cyan) is detected using a benchtop fluorescent microscope. C- Quantification of beta-galactosidase staining of cells in (A) 46 or 84 days after transduction. The graph represents the percentage of beta-galactosidase positive cells among the cell population. Statistical significance was determined using the one-way Anova test, n=2 (* indicates $p<0.05$). D- Growth curves of the indicated cell lines. The graph represents the cumulative PDs as a function of days of growth. E- Beta-galactosidase staining of HDF and HGPS cells before or after E6E7 expression. The staining was performed on freshly grown cells. The percentage of beta-gal positive cells among the cell population in each condition is indicated, n=2. F- FACS profiles of propodium Iodide (x-axis) and BrdU (y-axis) co-staining in the indicated cell lines. The percentage of cells in the different quadrants is indicated, with Q4 quadrant representing G0/G1, Q1-Q2 corresponding to S-phase and Q3 showing G2-M populations, n=2. G- Quantification of FACS profiles from (F). The percentage of cells in G0/G1, S and G2/M is shown. The BrdU signal was used to determine the percentage of cells in S phase. H- Quantification of LaminB1 intensity from immunostaining in the indicated cell lines. Mean+SD is shown, n=2. Statistical significance was determined using the Mann-Whitney test (**** indicates $p<0.0001$).

SUP. FIGURE 3

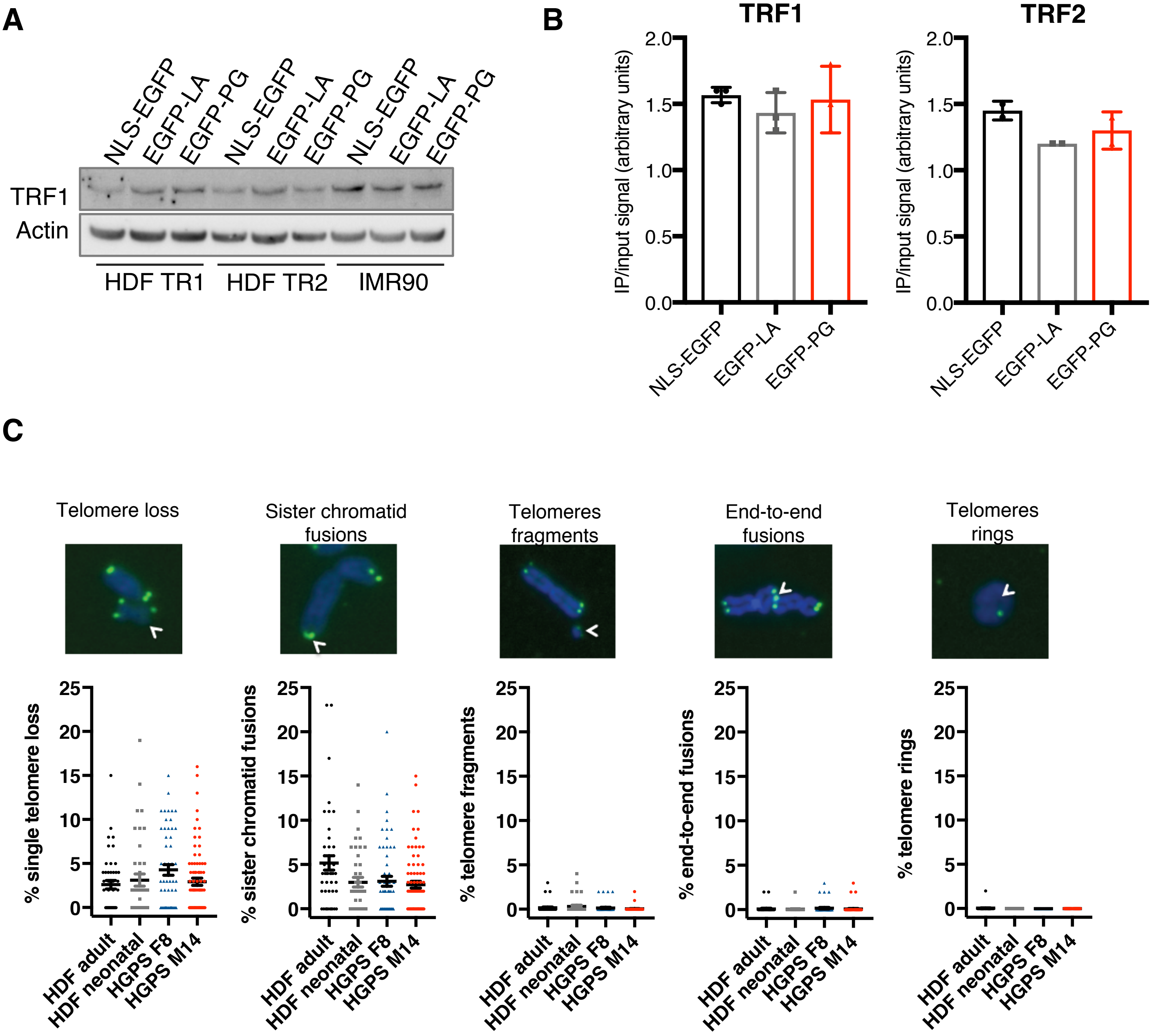

Supplementary Figure 3. Related to Figure 3

A- Western blot of the indicated cell lines (TR1 and TRF2 are independent transductions). Anti-TRF1 and anti-Actin signals are shown. B- Quantification of ChIPs shown in Fig. 3B. Each graph represents the enrichment in the IP samples normalized to the INPUT material. Mean intensities are shown ± SD (TRF1, n=3; TRF2, n=2 – except LA sample). C- Quantification of telomere aberrations (single telomere loss, sister chromatid fusion, telomere fragments, end-to-end fusions and telomere rings) scored on metaphase spreads from HGPS cells or controls as indicated. Telomeres were labeled by FISH with a FITC PNA probe. Representative images of each type of aberration are shown on top of each graph.

SUP. FIGURE 4

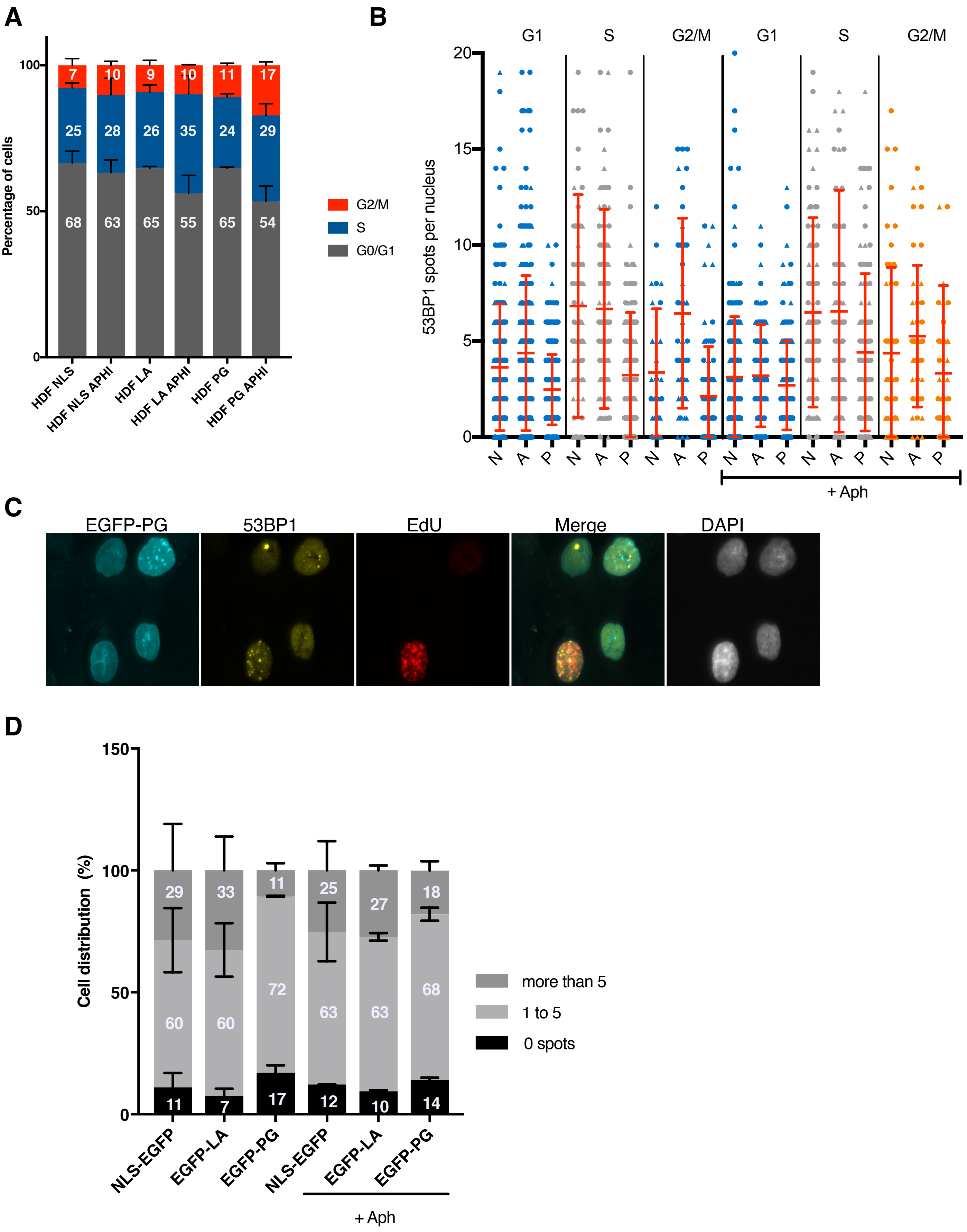

A
Percentage of cells
100
50
0
7
10
9
10
11
17
25
28
26
35
24
29
68
63
65
55
65
54
HDF NLS
HDF NLS APHI
HDF LA
HDF LA APHI
HDF PG
HDF PG APHI
G2/M
S
G0/G1
B
G1
S
G2/M
G1
S
G2/M
53BP1 spots per nucleus
20
15
10
5
0
N
A
P
N
A
P
N
A
P
N
A
P
N
A
P
N
A
P
+ Aph
C
EGFP-PG
53BP1
EdU
Merge
DAPI
D
Cell distribution (%)
150
100
50
0
29
33
11
25
27
18
60
60
72
63
63
68
11
7
17
12
10
14
more than 5
1 to 5
0 spots
NLS-EGFP
EGFP-LA
EGFP-PG
NLS-EGFP
EGFP-LA
EGFP-PG
+ Aph

Supplementary Figure 4. Related to Figure 4

A- Quantification of the percentage of cells in G0/G1, S and G2/M obtained from QIBC experiments performed on HDF cells expressing NLS-EGFP, EGFP-LA or EGFP-PG, before or after 0.2mM aphidicolin treatment. The percentage of cells in each phase in indicated on the graph. B- Quantification of QIBC for 53BP1 staining performed on HDF expressing NLS-EGFP (N), EGFP-LA (A) or EGFP-PG (P), treated or not with 0.2mM of aphidicolin as in Fig 4E. Nuclei were sorted in G1, S and G2/M according to the DAPI and EdU signals. Mean with SD is shown, n=2. C- Representative immunostaining of HDF cells expressing EGFP-PG acquired on a wide field microscope. EGFP (cyan), 53BP1 (Yellow), EdU (red) and DAPI is shown. D- Same quantification as in B, but represented as a percentage of cells with 0, 1 to 5, or more than 5, 53BP1 spots per nucleus.

SUP. FIGURE 5

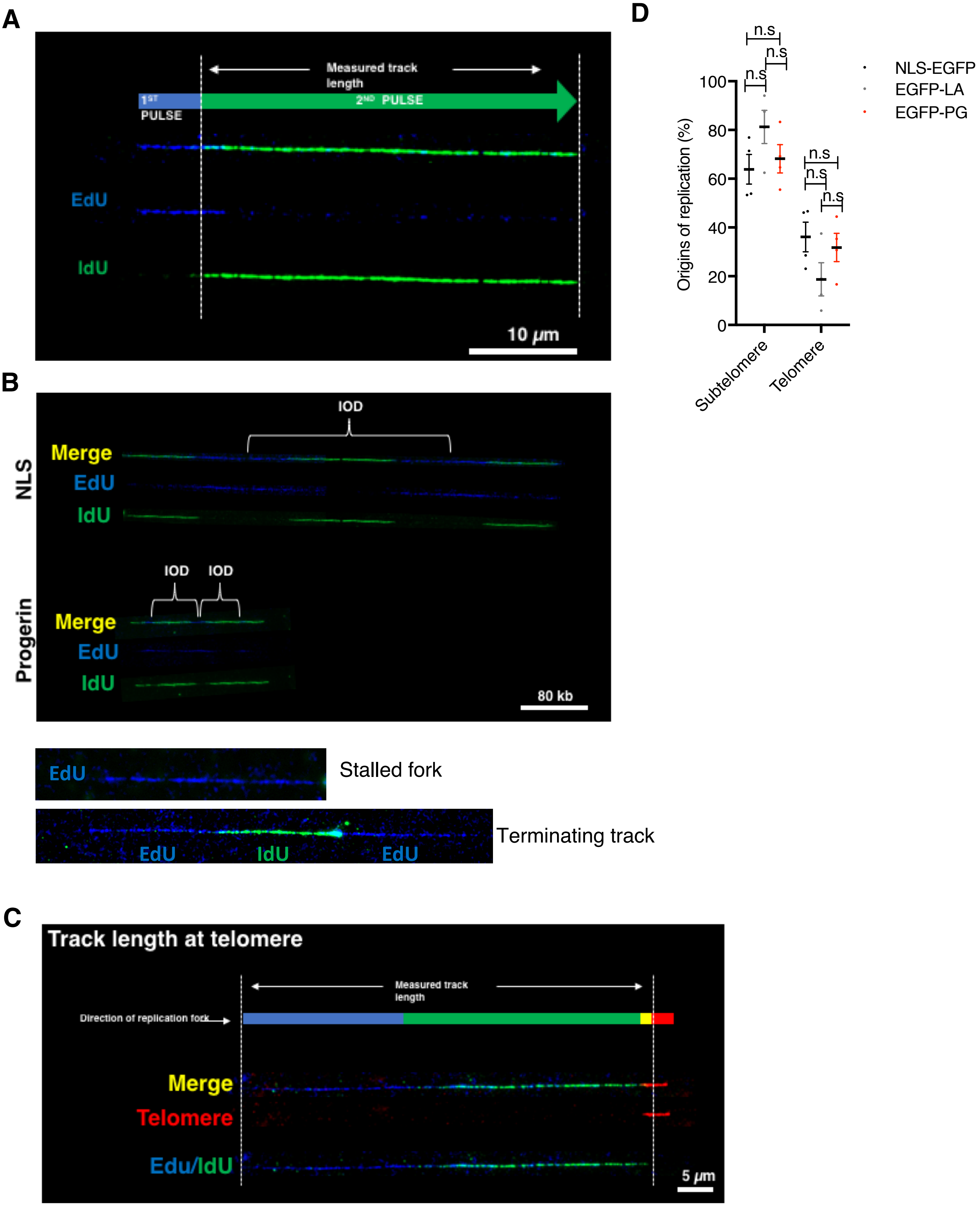
A
Measured track length
1ST PULSE
2ND PULSE
EdU
IdU
10 μm
B
IOD
NLS
Merge
EdU
IdU
IOD
IOD
Progerin
Merge
EdU
IdU
80 kb
EdU
Stalled fork
EdU
IdU
EdU
Terminating track
C
Track length at telomere
Measured track length
Direction of replication fork
Merge
Telomere
Edu/IdU
5 μm
D
n.s
n.s
n.s
n.s
n.s
n.s
100
80
60
40
20
0
Origins of replication (%)
Subtelomere
Telomere
NLS-EGFP
EGFP-LA
EGFP-PG

Supplementary Figure 5. Related to Figure 5

A- Representative example of measured EdU and IdU tracks length for the SMARD analysis. B- Representative example of measured Inter Origin Distance (IOD) for the SMARD analysis. One example is shown for HDF expressing NLS-EGFP, and one example for EGFP-PG. Below: example of a stalled fork with only EdU staining (first pulse), and of a terminating track (IdU signal framed by EdU signal). C- Representative example of measured EdU and IdU tracks length at telomeres for the SMARD analysis. DNA fibers with a terminal telomeric FISH signal (red) preceded by EdU and/or IdU signals are used. D- Quantification of the percentage of origins of replication at telomeric and sub-telomeric regions in HDF cells expressing NLS-EGFP, EGFP-LA, or EGFP-PG. Mean with SED is show, n=3. Statistical significance was determined using one-way Anova test.

SUP. FIGURE 6

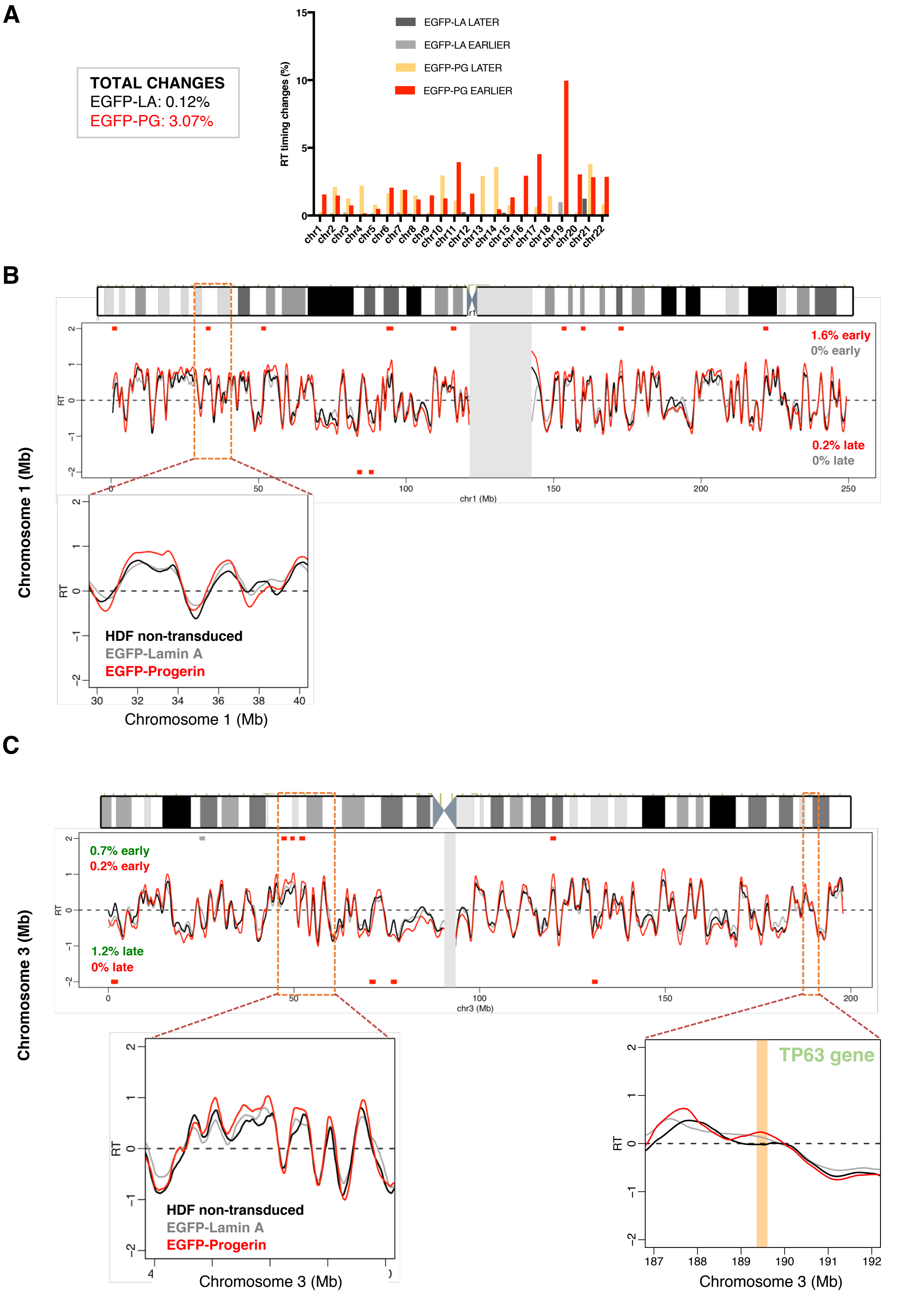
A
TOTAL CHANGES
EGFP-LA: 0.12%
EGFP-PG: 3.07%
EGFP-LA LATER
EGFP-LA EARLIER
EGFP-PG LATER
EGFP-PG EARLIER
RT timing changes (%)
chr1 chr2 chr3 chr4 chr5 chr6 chr7 chr8 chr9 chr10 chr11 chr12 chr13 chr14 chr15 chr16 chr17 chr18 chr19 chr20 chr21 chr22
B
Chromosome 1 (Mb)
1.6% early
0% early
0.2% late
0% late
chr1 (Mb)
RT
HDF non-transduced
EGFP-Lamin A
EGFP-Progerin
Chromosome 1 (Mb)
C
Chromosome 3 (Mb)
0.7% early
0.2% early
1.2% late
0% late
chr3 (Mb)
HDF non-transduced
EGFP-Lamin A
EGFP-Progerin
Chromosome 3 (Mb)
TP63 gene
Chromosome 3 (Mb)

Supplementary Figure 6. Related to Figure 6

A- Changes in RT profiles in HDFs expressing EGFP-LA or EGFP-PG compared to non-transduced cells. The graph represents changes to earlier or later timing for each chromosome. The percentage of total changes in the genome is also indicated. B&C- RT profiles of chromosome 1 (B) and 3 (C) for HDF cells non-transduced or expressing EGFP-LA or EGFP-PG. The graph displays the log2 ratio of signals from early and late S-phase fractions. Positive values correspond to early replication, and negative values correspond to late replication. The magnified area highlights chromosome segments showing RT alterations in progerin-expressing cells. Region of the chromosome 3 corresponding to the *TP63* gene from Rivera-Mulia et al is also shown.

SUP. FIGURE 7 – ORIGINAL BLOTS

*signal from previous staining

** aspecific

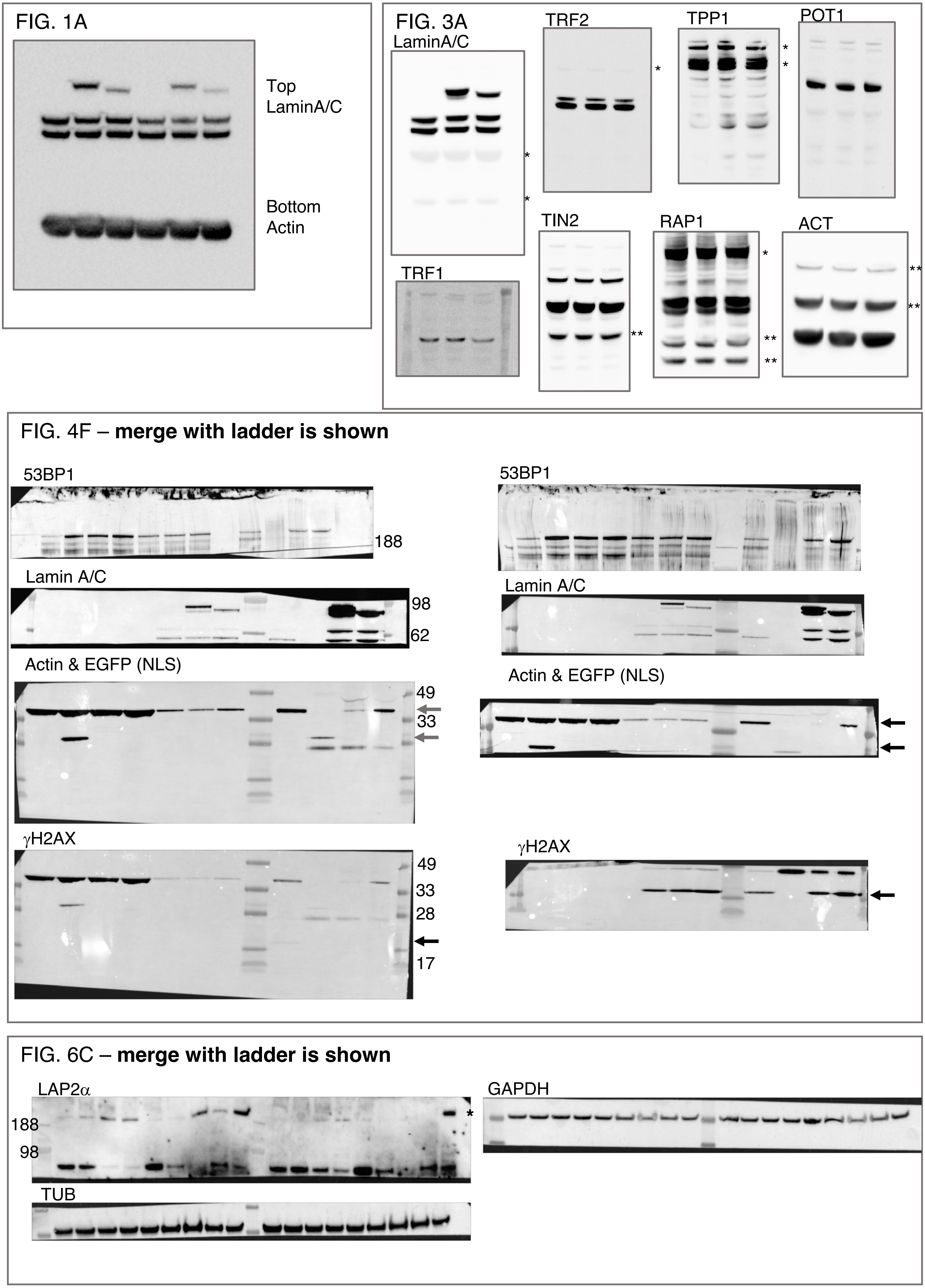

SUP. FIGURE 8 – ORIGINAL BLOTS

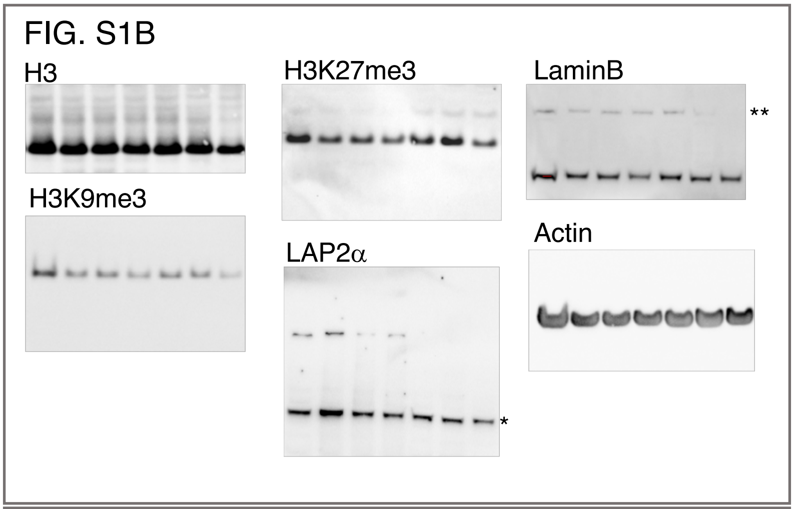

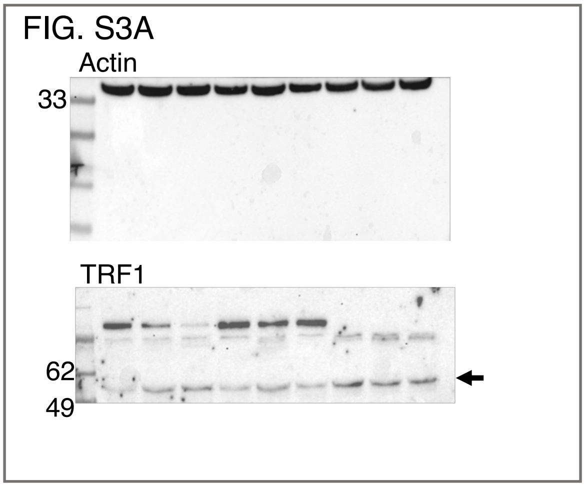

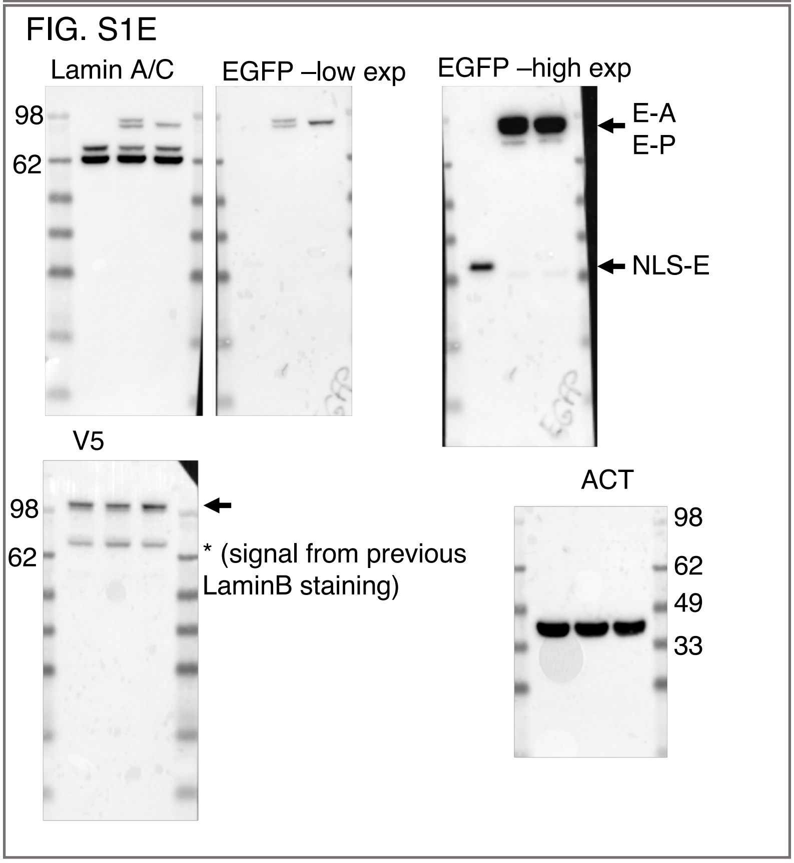

Supplement: Supplementary file 1 — Supplementary Information. [file 41598_2021_92631_MOESM1_ESM.pdf]
